# Marked oestrous cycle‐dependent regulation of rat arterial K_V_7.4 channels driven by GPER1

**DOI:** 10.1111/bph.15947

**Published:** 2022-10-11

**Authors:** Samuel N. Baldwin, Elizabeth A. Forrester, Natalie Z. M. Homer, Ruth Andrew, Vincenzo Barrese, Jennifer B. Stott, Brant E. Isakson, Anthony P. Albert, Iain A. Greenwood

**Affiliations:** ^1^ Vascular Biology Research Centre, Institute of Molecular and Clinical Sciences St George's University of London London UK; ^2^ Mass Spectrometry Core Laboratory, Edinburgh Clinical Research Facility, Queen's Medical Research Institute University of Edinburgh Edinburgh UK; ^3^ BHF Centre for Cardiovascular Science, Queen's Medical Research Institute University of Edinburgh Edinburgh UK; ^4^ Department of Neuroscience, Reproductive Sciences and Dentistry University of Naples Federico II Naples Italy; ^5^ Department of Molecular Physiology and Biophysics, Robert M. Berne Cardiovascular Research Centre University of Virginia School of Medicine Charlottesville Virginia USA

## Abstract

**Background and Purpose:**

*Kcnq*‐encoded K_V_7 channels (termed K_V_7.1–5) regulate vascular smooth muscle cell (VSMC) contractility at rest and as targets of receptor‐mediated responses. However, the current data are mostly derived from males. Considering the known effects of sex, the oestrous cycle and sex hormones on vascular reactivity, here we have characterised the molecular and functional properties of K_V_7 channels from renal and mesenteric arteries from female Wistar rats separated into di‐oestrus and met‐oestrus (F‐D/M) and pro‐oestrus and oestrus (F‐P/E).

**Experimental Approach:**

RT‐qPCR, immunocytochemistry, proximity ligation assay and wire myography were performed in renal and mesenteric arteries. Circulating sex hormone concentrations were determined by liquid chromatography–tandem mass spectrometry. Whole‐cell electrophysiology was undertaken on cells expressing K_V_7.4 channels in association with G‐protein‐coupled oestrogen receptor 1 (GPER1).

**Key Results:**

The K_V_7.2–5 activators S‐1 and ML213 and the pan‐K_V_7 inhibitor linopirdine were more effective in arteries from F‐D/M compared with F‐P/E animals. In VSMCs isolated from F‐P/E rats, exploratory evidence indicates reduced membrane abundance of K_V_7.4 but not K_V_7.1, K_V_7.5 and Kcne4 when compared with cells from F‐D/M. Plasma oestradiol was higher in F‐P/E compared with F‐D/M, and progesterone showed the converse pattern. Oestradiol/GPER1 agonist G‐1 diminished K_V_7.4 encoded currents and ML213 relaxations and reduced the membrane abundance of K_V_7.4 and interaction between K_V_7.4 and heat shock protein 90 (HSP90), in arteries from F‐D/M but not F‐P/E.

**Conclusions and Implications:**

GPER1 signalling decreased K_V_7.4 membrane abundance in conjunction with diminished interaction with HSP90, giving rise to a ‘pro‐contractile state’.

## Graphical abstract

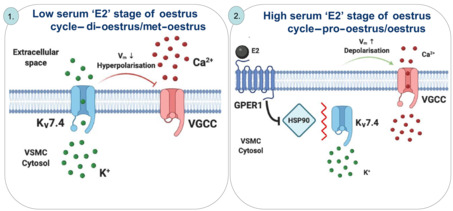

**Abbreviations**
BK_Ca_
: large conductive calcium activated potassium channel
E2: oestradiol
EC(−): endothelium denuded
EC(+): endothelium intact
ECs: endothelial cells
F‐D/M: di‐oestrus and met‐oestrus
F‐P/E: pro‐oestrus and oestrus
FSH: follicular stimulating hormone
GPER1: G‐protein‐coupled oestrogen receptor 1
HEK‐K_V_7.4: human embryonic kidney 293B stably expressing K_V_7.4
HEK‐K_V_7.4‐GPER1: HEK‐K_V_7.4 cells transiently transfected with *GPER1*
HSP90: heat shock protein 90
K^+^PSS: high K^+^ physiological salt solution
K_ATP_
: ATP‐sensitive potassium channel
K_V_
: voltage‐gated potassium channel
LC–MS/MS: liquid chromatography–tandem mass spectrometry
LH: luteinising hormone
PLA: proximity ligation assay
PSS: physiological salt solution
VGCC: voltage‐gated calcium channels
VSMCs: vascular smooth muscle cells

**What is already known**

- Vascular K_V_7 channels are key components of resting tone and endogenous vasoactive responses.
- The oestrous cycle regulates vascular reactivity.

**What does this study add**

- Oestrous cycle‐dependent reduction in K_V_7.4 membrane abundance and function coincided with a pro‐contractile phenotype.
- GPER1 activation negatively regulates K_V_7.4 forward trafficking and function.

**What is the clinical significance**

- Oestrogenic signalling decreases vascular K_V_7 channel function.
- Negative regulation of K_V_7.4 may contribute to the detrimental attributes of hormone replacement therapy.

## 1 INTRODUCTION

Sexual dimorphisms in cardiovascular physiology and pathophysiology are a well‐documented phenomenon (Pabbidi et al., 2018). Pre‐menopausal women exhibit greater coronary and cerebral blood flow, and the incidence of adverse cardiovascular events is significantly lower (Pabbidi et al., 2018). As the female cardioprotective phenotype decreases after the menopause, the most likely candidates that drive sexual dimorphisms within the vasculature are sex hormones, primarily oestrogens. However, the role of oestrogens within the cardiovascular system remains enigmatic, as they have been shown to be both protective and detrimental to the vasculature (Hulley et al., 1998, 2002; Yang & Reckelhoff, 2011).

Within rodent and human arteries, *KCNQ*‐encoded K_V_7 channels are key regulators of vascular reactivity, whereby activation of the channel mediates hyperpolarisation of the membrane and closure of voltage‐gated calcium channels (VGCC). In vascular smooth muscle, of the five subtypes, *KCNQ1* , *KCNQ4* and *KCNQ5* are the principally expressed genes (Ng et al., 2011; Ohya et al., 2003), K_V_7.4 is the predominantly expressed protein (Ng et al., 2011; Yeung et al., 2007) and the K_V_7.4/K_V_7.5 heterotetramer is purported to be the most common channel species (Chadha et al., 2014). Pharmacological and molecular evidence shows that activity of K_V_7.4/K_V_7.5 channels regulates resting membrane potential (Mackie et al., 2008) and is functionally important for cAMP‐ and cGMP‐linked receptor‐mediated vasorelaxation (Chadha et al., 2012, 2014; Mondéjar‐Parreño et al., 2019; Stott et al., 2015) and PKC‐mediated contraction (Brueggemann et al., 2006). Notably, K_V_7.4 channels are down‐regulated in hypertensive rats (Jepps et al., 2011) via post‐transcriptional mechanisms affecting protein synthesis, trafficking and degradation (Barrese, Stott, Figueiredo, et al., 2018; Carr et al., 2016) and are associated with the hypertensive phenotype (Barrese, Stott, & Greenwood, 2018).

To date, data on vascular K_V_7 channels focuses primarily on arteries from male animals. On the basis of known sexual dimorphisms in vascular reactivity and the growing recognition of sex as an experimental factor (Docherty et al., 2019), we aimed to address this deficit. We have characterised the functional and molecular properties of K_V_7 channels in arteries from female Wistar rats. In light of previously demonstrated oestrous cycle‐dependent changes in vascular reactivity (Jaimes et al., 2019), the oestrous cycle was a key consideration in the following study. The rat oestrous cycle lasts only 4–5 days, with different durations for each stage, as follows: (1) pro‐oestrus, 14 h; (2) oestrus, 24–48 h; (3) met‐oestrus, 6–8 h; and (4) di‐oestrus, 48–72 h (Cora et al., 2015). As sex hormones peak in pro‐oestrus (Nilsson et al., 2015), female rats were separated into two groups, those in pro‐oestrus and oestrus (F‐P/E) and those in di‐oestrus and met‐oestrus (F‐D/M) stages of the oestrous cycle. The observations detailed here demonstrate a remarkable oestrous cycle‐related reduction in K_V_7.4 membrane abundance, mediated through oestradiol (E2) signalling via G‐protein‐coupled oestrogen receptor 1 (GPER1), which underlies a pro‐contractile vascular state.

## 2 METHODS

### 2.1 Animal model

All animal care and experimental procedures complied with the requirements of the UK Animal (Scientific Procedures) Act (ASPA) 1986 and were approved by the Institutional Animal Ethics Committee. Animal studies are reported in compliance with the ARRIVE guidelines (Percie du Sert et al., 2020) and with the recommendations made by the *British Journal of Pharmacology* (Lilley et al., 2020). Experiments were performed with male and female Wistar rats (RRID:RGD_734476; Charles River, Margate, UK) aged 11–15 weeks (200–300 g) kept at the Biological Research Facility at St George's University (London). The animals were housed in cages with free access to water and food (RM1; Dietex International, UK) on a 12‐h light/dark cycle and maintained at a constant temperature and humidity (21°C ± 1°C; 50% ± 10% humidity) in accordance with the ASPA 1986. Animals were kept in a bedding of LSB Aspen woodchip. Female rats were housed separately from males to ensure standard progression through the oestrous cycle. Animals were killed humanely by cervical dislocation with secondary confirmation via femoral artery severance, in accordance with Schedule 1 of the ASPA 1986. Organs were harvested and immediately placed in ice‐cold physiological salt solution (PSS) of the following composition (mmol·L^−1^): 119 NaCl, 4.5 KCl, 1.17 MgSO_4_·7H_2_0, 1.18 NaH_2_PO_4_, 25 NaHCO_3_, 5 glucose and 1.25 CaCl_2_ balanced to a pH of 7.4 by perfusion of 95% O_2_ and 5% CO_2_. Blood (2 ml) was collected during femoral artery severance, into vials containing EDTA (100 μl of 0.5M in H_2_O). Samples were then centrifuged at 2000 x *g* for 20 min. Plasma was extracted and stored at −80°C.

### 2.2 Oestrous cycle stage determination

Following humane killing, PSS (50 μl ) was inserted into the vaginal canal via a 2–200 μl tip and flushed 4–6 times to liberate cells from the surface of the cervix. PSS was removed from the vaginal canal, and 25 μl of the cervical cell suspension was mounted on a glass slide and examined under light microscopy. Variation in the population of three principal cell types ‐ large keratinised (cornified) epithelial cells, nucleated epithelial cells and leukocytes ‐ were used to identify each stage, as previously described (Cora et al., 2015), which was the primary tool used for cycle stage determination during the course of this study. Representative images from each cycle stage are shown in Figure S1. To generate the representative images in Figure S1, samples of cervical cell suspension (50 μl) were plated on glass slides and left to adhere for 1 h at room temperature. Then the slide was flooded with toluidine blue O (1ml, 0.1% in H_2_O, passed through a 0.2‐μm syringe filter). Cells were left in the dye solution for 45 s, before being washed in distilled water on a rotating plate (20 rpm) for 1 min. Cells were left to dry and then imaged via Nikon Eclipse Ni. Cycle stage determination was performed after the experiment, during functional investigation, as a means of blinding. Such blinding was not possible during molecular techniques.

### 2.3 Wire myography

Arterial segments (~2 mm) of the main renal, second‐order mesenteric, basilar and left anterior descending coronary arteries were mounted on either 100 μm pins (renal) or 40 μm tungsten wire (mesenteric, coronary and cerebral arteries) within a myograph chamber (Danish Myo Technology, Arhus, Denmark) containing PSS (5 ml) oxygenated with 95% O_2_ and 5% CO_2_ at 37°C. Vessels then underwent a passive force normalisation process to achieve an internal luminal circumference at a transmural pressure of 100 mmHg (13.3 kPa) to standardise pre‐experimental conditions (Mulvany & Halpern, 1976). Force generated was amplified by a PowerLab (ADInstruments, Oxford, UK) and then recorded via LabChart software (RRID:SCR_017551; ADInstruments). Vessels were then left to rest for 10 min. A minimal time interval that was applied between all separate challenges to the vessels. Isotonic high K^+^ physiological salt solution (K^+^PSS) of the following composition (mmol·L^−1^): 63.5 NaCl, 60 KCl, 1.17 MgSO_4_·7H_2_0, 1.18 NaH_2_PO_4_, 25 NaHCO_3_, 5 glucose and 1.25 CaCl_2_ balanced to a pH of 7.4 by gassing with 95% O_2_ and 5% CO_2,_ was then added to bath to determine vessel viability. After the contraction had stabilised, the vessels were washed in normal PSS until they returned to baseline. Vessels were then challenged again with K^+^PSS to ensure maximal contraction had been achieved. Endothelial cell (EC) integrity was determined by relaxation of pre‐constricted arterial tone (methoxamine, 10 μmol·L^−1^) in response to carbachol (10 μmol·L^−1^). Only vessels that generated ≥80% relaxation were used and considered endothelium‐positive.

When generating concentration–effect curves in response to the TXA_2_ receptor agonist U46619 (0.003–3 μmol·L^−1^), logarithmically increasing concentrations of an agent were added to the bath following the ‘warm‐up’ protocol, with incremental increase in tension allowed to plateau before the next concentration was added. Upon completion of the curve, vessels were washed in standard PSS and allowed to return to baseline tension. Vessels were then pre‐incubated in either DMSO (≤0.1%) as the solvent control, linopirdine (10 μmol·L^−1^) or HMR‐1556 (10 μmol·L^−1^) for 10 min, before starting a second concentration–effect curve. All contractions were then normalised to the peak, stable contraction generated in response to K^+^PSS. In contrast, when investigating vasorelaxants, vessels were first pre‐constricted with U46619 (300 nmol·L^−1^). Once tone had stabilised, logarithmically increasing concentrations of either isoprenaline (0.003–3 μmol·L^−1^), S‐1 (0.1–10 μmol·L^−1^), ML213 (0.01/0.1–10 μmol·L^−1^), NS11021 (0.1–30 μmol·L^−1^), pinacidil (0.1–30 μmol·L^−1^) or nicardipine (0.001–1 μmol·L^−1^) were then added to the bath. With regard to isoprenaline, vessels were then washed and pre‐incubated in either DMSO (≤0.1%), linopirdine (10 μmol·L^−1^) or HMR‐1556 (10 μmol·L^−1^) for 10 min, and a second curve was generated. With experiments involving vasorelaxation in response to ion channel modulators, only one curve could be generated as these drugs do not readily wash out. EC denudation was achieved by mechanical abrasion of the lumen of the arterial segment with human hair and was validated by loss of the relaxation of pre‐contracted arterial tone (10 μmol·L^−1^ methoxamine) in response to carbachol.

### 2.4 RT‐qPCR procedures

Relative fold changes in expression levels of *Kcnq1–5*, *Kcne1–5*, *Esr1–2, Gper1, Acta2 and Cd31* transcripts were determined in main renal and mesenteric arteries and whole brain, heart and uterine samples via RT‐qPCR. In addition, endothelium intact (EC(+)) and endothelium denuded (EC(−)) lysates of mesenteric arteries were prepared. EC(−) samples were prepared as previously described (Askew Page et al., 2019).

mRNA was extracted and converted to cDNA using Monarch Total RNA Miniprep Kit (New England BioLabs, Ipswich, MA, USA) then LunaScript RT SuperMix Kit (New England BioLabs), respectively. Quantitative analysis of target genes was assessed via CFX‐96 Real‐Time PCR Detection System (RRID:SCR_018064; BioRad, Hertfordshire, UK). Samples were run in BrightWhite qPCR plates (Primer Design, Camberley, UK) in combination with PrecisionPLUS qPCR Master Mix (Primer Design), 300 nmol·L^−1^ of gene‐specific target primer (Thermofisher Scientific, Waltham, MA, USA) and 10 ng cDNA, according to the manufacturer's instructions. Quantification cycles (Cq) were determined via Bio‐Rad CFX96 Manager 3.0. Cq was normalised to the average of two housekeeping genes, chosen from ubiquitin C (*Ubc*), polyamine transporter 1 (*Tpo‐1*), cytochrome C1 (*Cyc1*), calnexin (*Canx*) and glyceraldehyde 3‐phosphate dehydrogenase (*Gapdh*) and expressed using either formula 2^−ΔCq^ or 2^−ΔΔCq^ for analysis of relative abundance or relative fold changes as stated (Jepps et al., 2011). A list of the primers used (Thermo‐Fisher, Paisley, UK) in the experiments described here is given in Table 1. Primers for housekeeping genes were provided by Primer Design; such sequences cannot be disclosed for proprietary reasons.

**TABLE 1:** Primers used in RT‐qPCR analyses

Gene	(+) Forward primer sequence	Gene accession number	Amplicon (bp)
	(−) Reverse primer sequence		
*Kcnq1*	TGGGTCTCATCTTCTCCTCC	NM_032073	124
	GTAGCCAATGGTGGTGACTG		
*Kcnq2*	AAGAGCAGCATCGGCAAAAA	NM_133322	101
	GGTGCGTGAGAGGTTAGTAGCA		
*Kcnq3*	CAGCAAAGAACTCATCACCG	AF091247	161
	ATGGTGGCCAGTGTGATCAG		
*Kcnq4*	GAATGAGCAGCTCCCAGAAG	XM_233477.8	133
	AAGCTCCAGCTTTTCTGCAC		
*Kcnq5*	AACTGATGAGGAGGTCGGTG	XM_001071249.3	120
	GATGACCGTGACCTTCCAGT		
*Kcne1*	GTTTCCCCAAATCTCTCCATT	NM_008424.3	111
	AGCACACACTTCCCATTTCAA		
*Kcne2*	CCTGGTATTTAACTGAGTTGGACAT	NM_133603.2	97
	GCACTGGGGGCTCTTGAAT		
*Kcne3*	CTCAACCATATCAAGCCACAGT	NM_022235.2	99
	GCCTATCAGTCCCTCTTCTCT		
*Kcne4*	GGAGGAGGGGGCTGATGA	NM_212526.1	88
	CTGGTGGATGTTCTCGGAAGA		
*Kcne5*	GCACGAAGAGACCTCAGACAT	NM_001101003.1	146
	GGACAGGAAACAAGAACACCAT		
*Esr1*	TTCACCTTCTGGAGTGTGCC	NM_012689.1	173
	ACTTGACGTAGCCAGCAACA		
*Esr2*	TGCCGACTTCGCAAGTGTTA	NM_012754.2	138
	ACCGTTTCTCTTGGCTTTGC		
*Gper1*	TCATCGGCCTGTGCTATTCC	NM_133573	119
	GAAGACAAGGACCACTGCGA		
*Cd31*	CTCCTAAGAGCAAAGAGCAACTTC	NM_031591.1	100
	TACACTGGTATTCCATGTCTCTGG		
*Acta2*	ATCCGATAGAACACGGCATC	NM_031004.2	228
	AGGCATAGAGGGACAGCACA		

### 2.5 Vascular smooth muscle cell isolation

Renal and mesenteric arteries were incubated in isolation PSS of the following composition (mmol·L^−1^): 120 NaCl, 6 KCl, 12 glucose, 10 HEPES and 1.2 MgCl_2_ balanced to a pH of 7.4 with NaOH, supplemented with 1.75 mg·ml^−1^ collagenase type IA, 0.9 mg·ml^−1^ protease, 1 mg·ml^−1^ trypsin inhibitor and 1 mg·ml^−1^ bovine serum albumin (Sigma, UK) at 37°C for 30 min (renal artery) or 17 min (mesenteric artery). Next, vessels were gently triturated with a wide‐bore glass pipette to liberate vascular smooth muscle cells (VSMCs) from their extracellular matrix. The subsequent cell suspension was supplemented with 2.5 mmol·L^−1^ Ca^2+^ and left to adhere on 25 mm glass coverslips for 1 h in an incubator at 37°C in 95% O_2_ + 5% CO_2_. Isolated myocytes were then either fixed immediately afterwards or incubated in 1 ml isolation PSS containing solvent control (DMSO or ethanol, final solvent concentrations were ≤0.1%), or the following ‐ oestradiol (E2; 10 nmol·L^−1^), G‐36 (1 μmol·L^−1^) or G‐1 (1 μmol·L^−1^) for 10 or 30 min as stated.

### 2.6 Immunocytochemistry

The immuno‐related procedures used comply with the recommendations made by the *British Journal of Pharmacology* (Alexander et al., 2018). Isolated VSMCs were fixed in 3% paraformaldehyde (PFA) containing phosphate buffered serum (PBS) for 15 min at room temperature (Barrese, Stott, Figueiredo, et al., 2018). For membrane staining, cells were incubated in Alexa Fluor 488‐conjugated wheat germ agglutinin (WGA; Thermo‐Fisher; dil. 1:200 in PBS) for 10 min, washed in 0.1 mmol·L^−1^ glycine in PBS for 5 min and incubated in blocking solution (0.1% Triton X‐100, 10% fetal bovine serum in PBS) for 1 h. Cells were incubated overnight in either rabbit anti‐K_V_7.4 (RRID:AB_2341042; #APC‐164; Alomone, Jerusalem, Israel; dil. 1:200), rabbit anti‐K_V_7.1 (Pineda, Antikörber‐Service, Germany; dil. 1:100), rabbit anti‐K_V_7.5 (RRID:AB_210806; #ABN1372; Millipore, Temecula, CA, USA; dil. 1:100) or rabbit anti‐Kcne4 (RRID:AB_1079170; #HPA011420; Atlas Antibodies, Sweden; dil. 1:200) at 4°C. Cells were then washed in PBS and incubated in goat anti‐rabbit secondary antibody conjugated to Alexa Fluro 568 (RRID:AB_143157; A11036; Thermo‐Fisher; dil. 1:100), and then mounted in Vectashield (P4170; Sigma) medium containing 4′,6‐diamidino‐2‐phenylindole (DAPI). All antibodies were diluted in blocking solution. Cells were then imaged via Nikon A1R confocal microscope (inverted) on Ti2 chassis (Image Resource Facility, St George's University, London). For experiments determining the membrane:cytosol ratio for K_V_7.4 expression, fluorescence intensity profiles for K_V_7.4 and WGA were plotted across three randomly drawn lines spanning the width of the cell measured in arbitrary units (A.U.) using ImageJ software (RRID:SCR_003070; https://imagej.nih.gov/ij/). Fluorescence intensity ≥ 200 A.U. was considered as the plasma membrane, and below the threshold was considered as the cytosol. The membrane:cytosol ratio for K_V_7.4 expression was calculated by measuring the average fluorescence intensity of K_V_7.4 within the membrane and dividing it by the average fluorescence intensity of K_V_7.4 within cytosol in 10–12 cells per n. Total cell fluorescence was measured by via ImageJ software (https://imagej.nih.gov/ij/). Validation of rabbit anti‐K_V_7.4 (#APC‐164) is demonstrated in Figure S2.

### 2.7 Plasma concentration of sex hormones

Steroid analysis was performed by targeted liquid chromatography–tandem mass spectrometry (LC–MS/MS), following extraction of samples through automated supported liquid extraction (SLE) on an Extrahera liquid handling robot (Biotage, Uppsala, Sweden), adapted from Boulton et al. (2021). The method has an intra‐ and inter‐assay coefficient variation between 4.9% and 7.2% for the five steroids measured: E2, aldosterone, testosterone, androstenedione and progesterone. Analysis was performed on an I‐class Acquity UPLC (Waters, Wilmslow, UK) interfaced to a QTRAP 6500+ (AB Sciex, Warrington, UK) mass spectrometer. Instrument control and data acquisition were achieved using Analyst® 1.6.3 Software. Data were integrated and evaluated using MultiQuant® 2.3.1 (AB Sciex). Chromatographic separation was achieved on a Kinetex C18 (2.1 × 150 mm; 2.6 μm particle size), column fitted with a KrudKatcher Ultra In‐Line Filter (0.5 μm porosity) both from Phenomenex, UK. The mobile phase system comprised mobile phase A ‐ water with ammonium fluoride (50 μM) and mobile phase B – methanol with ammonium fluoride (50 μM) pumped at a flow rate of 0.3 ml·min^−1^ over 16 min, starting at 55% B for 2 min, rising to 100% B over 6 min, held for 2 min, before returning to 55% B over 0.1 min and equilibrating for 4.9 min, all held at a temperature of 50°C. The solvent flow was diverted to waste from 0 to 2 min and 11 to 16 min.

The mass spectrometer was operated in electrospray ionisation mode with polarity switching using a TurboIonSpray source, and data were collected in unit resolution (0.7 *m/z* full width at half maximum). The source was operated at 600°C with polarity switching with an IonSpray voltage of 5.5 kV/−4.5 kV, a curtain gas of 30 psi, nitrogen nebuliser ion source gas 1 and heater ion source gas 2 of 40 and 60 psi, respectively. Multiple reaction monitoring transitions for steroids and their isotopically labelled internal standards are as follows with chromatographic retention time: negative ions following −4.5 kV IonSpray voltage for 17β‐oestradiol (7.0 min) *m/z* 271.0 ➔ 144.9, 182.9 at −21 and −19 V, ^13^C_3_‐[2,3,4]‐oestradiol (7.0 min) *m/z* 274.0 ➔ 147.9 at −29 V, aldosterone (2.6 min) *m/z* 359.1 ➔ 188.9, 331.0 at −21 and −35 V and d8‐aldosterone (2.6 min) *m/z* 367.2 ➔ 193.9 at −21 V; and positive ions for testosterone (7.6 min) *m/z* 289.1 ➔ 97.0, 109.2 at 12 and 6 V, ^13^C_3_‐[2,3,4]‐testosterone (7.6 min) *m/z* 292.1 ➔ 100.0 at −12 V, androstenedione (6.8 min) *m/z* 287.1 ➔ 97.0, 78.9 at 14 and 10 V, ^13^C_3_‐[2,3,4]‐androstenedione (6.8 min) *m/z* 290.2 ➔ 100.1 at −14 V and progesterone (8.9 min) *m/z* 315.0 ➔ 97.1, 109.1 at 25 and 27 V and 2,2,4,6,6,17α,21,21,21‐d9‐progesterone (8.9 min) *m/z* 324.1 ➔ 100.0, 109.1 at 15 V.

Calibration ranges (between 0.0025 and 100 ng·ml^−1^) for each steroid were plotted as the peak area ratio of the analyte divided by the relevant internal standard versus amount of steroid. Amounts of steroid were calculated using the calibration lines of best fit, which were considered acceptable if the regression coefficient, r, was >0.99, with 1/x weighting.

Plasma concentrations of luteinising hormone (LH) and follicular stimulating hormone (FSH) were determined at the University of Virginia Ligand Core for clinical and basic scientific research. Hormones were analysed via an own in‐house enzyme‐linked immunoblot assay (ELISA) protocol. LH was measured in plasma by a two‐step sandwich immunoassay using monoclonal antibodies against bovine LH (no. 581B7) and against the human LH‐β subunit (no. 5303: Medix Kauniainen, Finland) as described previously (Haavisto et al., 1993). FSH was assayed by RIA using mouse FSH reference prep AFP5308D for assay standards and mouse FSH antiserum (guinea pig; AFP‐1760191). See https://med.virginia.edu/research-in-reproduction/ligand-assay-analysis-core/ for further detail.

### 2.8 Single‐cell electrophysiology

Human embryonic kidney 293B (HEK293B) cells stably expressing human K_V_7.4 (HEK‐K_V_7.4; Copenhagen, Denmark) were grown in Dulbecco's modified Eagle's medium (DMEM)/F‐12 (Sigma) supplemented in 1% penicillin/streptomycin in 5% CO_2_ at 37°C. HEK‐K_V_7.4 cells were transiently transfected with *GPER1* (HEK‐K_V_7.4‐GPER1; HG11264‐ACG; pCMV3‐GPER1‐GFPSpark; 4 μg; Sino Biological, Eschborn, Germany) via Lipofectamine 3000 reagent (ThermoFisher Scientific), according to the manufacturer's instructions. HEK‐K_V_7.4‐GPER1 and same‐day non‐transfected controls were mounted on glass coverslips and left to attach for 1 h at room temperature. Cells were then incubated in either solvent, E2 (10 nmol·L^−1^) or G‐1 (1 μmol·L^−1^), for 30 min prior to generating ruptured whole‐cell current recordings.

Coverslips were mounted on an inverted microscope fitted with a Nikon C‐SHG mercury lamp. Cells were bathed in an external solution composed of (in mmol·L^−1^) 140 NaCl, 4 KCl, 2 CaCl_2_, 10 HEPES and 1 MgCl_2_ balanced to a pH of 7.4 with NaOH at room temperature. Glass pipettes (Plowden & Thompson) were pulled in‐house via PP‐830 (Narishige, Japan) to a resistance of 6–10 MΩ. Pipettes were filled with an internal solution composed of (in mmol·L^−1^) 110 K gluconate, 30 KCl, 0.5 MgCl, 5 HEPES, 0.5 EGTA and 1 Na_2_ATP balanced to a pH of 7.2 with CsOH. All currents in the following investigation were recorded using an AXOpatch 200B amplifier (Axon Instruments), and whole‐cell electrical signals were made and digitised via Digidata 1550A series operated by pClamp 10.7 (Molecular Devices). After membrane rupture, cells were held at −50 mV and pulsed to +20 mV every 20 s for 200 ms. Once currents had stabilised, current voltage relationships (*IV*) were constructed by stepping from −50 mV to ‘test voltages’ ranging from −70 to +40 mV for 1.5 s. Peak current amplitude normalised to cell size, *I*_(pA/pF)_, was measured following plateau at each voltage step. Voltage was stepped down to an inactivation step of ‐40 mV after each test and measured as a ‘tail current’.

### 2.9 Proximity ligation assay

The interaction of K_V_7.4 and heat shock protein 90 (HSP90) was determined by proximity ligation assay (PLA), as described by Barrese, Stott, Figueiredo et al., (2018). Mesenteric VSMCs were isolated, incubated in either ethanol (≤0.1%) or E2 (10 nmol·L^−1^), diluted in 1 ml of isolation PSS for 30 min at 37ºC and fixed as above. Cells were washed in 0.1 mmol·L^−1^ glycine containing PSS for 10 min, permeabilised via 0.1% Triton X‐100 for 5 min and blocked via Duolink blocking solution for 1 h at 37°C, and then incubated in a combination of rabbit anti‐K_V_7.4 (APC‐164, Alomone; dil. 1:200) and mouse‐anti‐HSP90 (RRID:AB_300396; ab13492, Abcam, Cambridge, UK; dil. 1:200) overnight at 4°C. The following morning, cells were incubated in a combination of Duolink In situ PLA probes, anti‐mouse MINUS (RRID:AB_2713942; DUO92004; Sigma‐Aldrich, St. Louis, MO, USA) and anti‐rabbit PLUS (RRID:AB_2810940; DUO92002; Sigma‐Aldrich) for 1 h at 37°C, according to the manufacturer's instructions. Using Duolink In situ detection reagents (DUO92008; Sigma‐Aldrich), samples underwent ligation (30 min at 37°C) and amplification (100 min at 37°C), according to the manufacturer's instructions. Cells were then mounted on cover slides in Vectashield (P4170; Sigma) containing DAPI. All antibodies and probes were diluted in blocking solution. Cells were then imaged in the Image Resource Facility, St George's University, London.

### 2.10 Data analysis

Figures show mean data from at least five repeats ± SEM. For functional experiments involving cumulative concentrations, a transformed data set was generated using X = Log(X) to reduce representative skew, following which a four‐parametric linear regression analysis was performed using the following equation: (Log (Agonist) vs. response − Variable slope (four parameters bottom/Hillslope/top/EC_50_)), using GraphPad Prism (RRID:SCR_002798; Version 8.2.0) to fit a concentration–effect curve to the figure. For data comparing multiple groups, a one‐way analysis of variance (ANOVA) was performed, or two‐way ANOVA followed by a post hoc Bonferroni/Dunnett's test to account for type 1 errors in multiple comparisons was performed for comparison of mean values. Multiple comparisons include condition A versus condition B at varying concentrations. Values of *P* ≤ 0.05 were considered to show significant effects. Some groups compared were of unequal numbers because of technical failure or an artefact of cycle stage determination, after the experiment, during functional investigations. Immunocytochemistry data was derived from 10‐15 cells from only 3 animals, therefore statistical analysis could not be performed. The data and statistical analysis comply with the recommendations of the *British Journal of Pharmacology* on experimental design and analysis in pharmacology (Curtis et al., 2018).

### 2.11 Materials

The reagents used in the present study were S‐1 and ML213, as specific activators of K_V_7.2–5 channels (Baldwin et al., 2020; Jepps et al., 2014); the pan‐K_V_7 channel blocker, linopirdine (10 μmol·L^−1^; Schnee & Brown, 1998); HMR‐1556, the specific blocker of K_V_7.1 channels (Thomas et al., 2003); the activator of large conductance calcium‐activated calcium channels (BK_Ca_), NS11021 (0.1–10 μmol·L^−1^); the activator of ATP‐sensitive potassium channel (K_ATP_), pinacidil (0.1–10 μmol·L^−1^); the inhibitor of VGCC channels, nicardipine (0.001–1 μmol·L^−1^); U46619 (0.003–3 μmol·L^−1^); β‐adrenoceptor agonist isoprenaline (0.003–3 μmol·L^−1^); E2 (0.01 μmol·L^−1^); the specific GPER1 agonist G‐1 (Dennis et al., 2009); and the specific GPER1 antagonist G‐36 (Dennis et al., 2011). All drugs for isometric tension recordings were obtained from Tocris Bioscience (Oxford, UK) except for S‐1 which was obtained from NeuroSearch (Ballerup, Denmark) and E2, which was purchased from Sigma‐Aldrich. Drugs were dissolved in DMSO or ethanol (E2), and diluted in PSS during isometric tension recording, isolation PSS during prior to cell fixing for immunocytoschemistry or external solution during single cell electrophysiology, final vehicle concentrations were ≤0.1%.

### 2.12 Nomenclature of targets and ligands

Key protein targets and ligands in this article are hyperlinked to corresponding entries in http://www.guidetopharmacology.org and are permanently archived in the Concise Guide to PHARMACOLOGY 2021/22 (Alexander, Christopoulos et al.; 2021; Alexander, Cidlowski et al., 2021; Alexander, Fabbro et al., 2021; Alexander, Mathie et al., 2021).

## 3 RESULTS

### 3.1 Oestrous cycle‐dependent changes in sensitivity to K^+^ channel modulators

**FIGURE 1:**
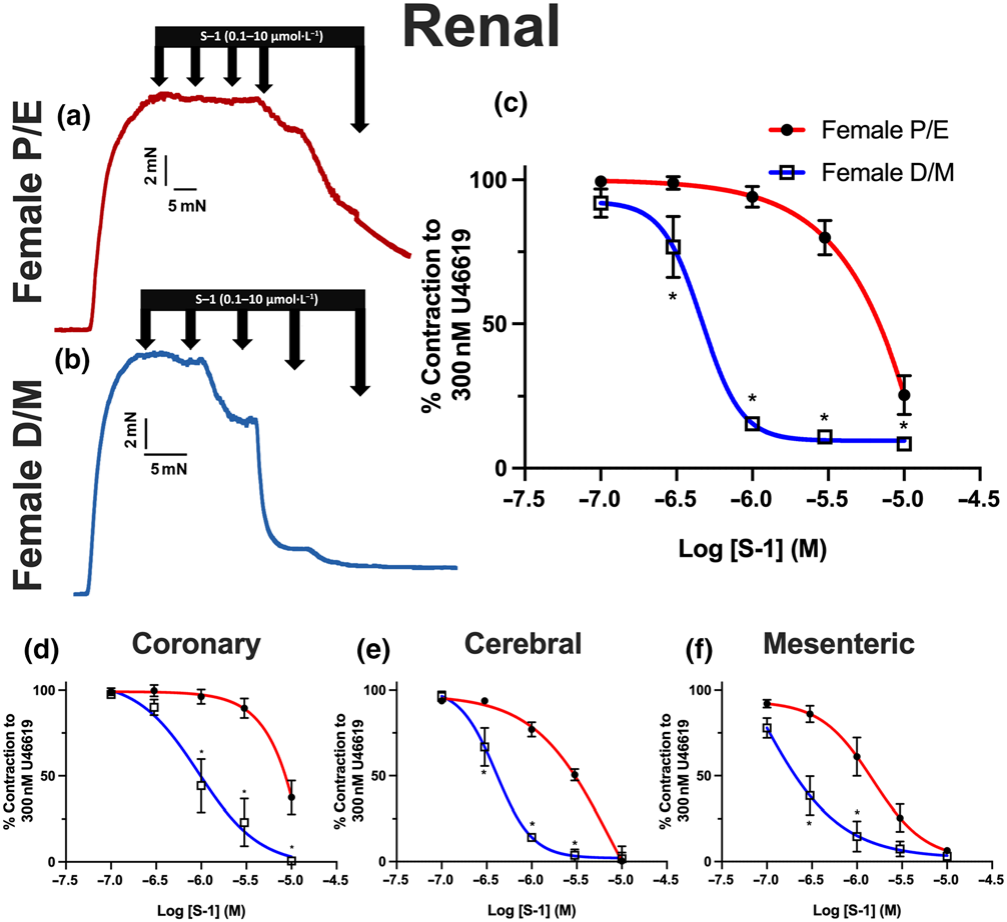
Oestrous cycle‐dependent differences in S‐1‐mediated relaxation of pre‐contracted tone within arteries from female (P/E2) and female (D/M) Wistar rats. Representative traces of relaxation of pre‐contracted arterial tone (U46619; 300 nmol·L^−1^) in renal arteries from female pro‐oestrus/oestrus (P/E; a) and female di‐oestrus/met‐oestrus (D/M; b) in response to K_V_7.2–5 activator S‐1 (0.1–10 μmol·L^−1^)*.* Mean data of S‐1‐mediated relaxation in renal (n = 5–8; c), coronary (n = 4–8; d), cerebral (n = 5–7; e) and mesenteric (n = 5–6; f) arteries. Data shown are means ± SEM; n = number of animals (c–f). *P ≤ 0.05, significantly different from female P/E; two‐way ANOVA with post hoc Bonferroni test.

The K_V_7.2–5 channel activator S‐1 (0.1–10 μmol·L^−1^) evoked concentration‐dependent relaxation of pre‐constricted arterial tone (300 nmol·L^−1^ U46619) in arteries from both F‐D/M and F‐P/E (see representative traces in Figure 1a,b). S‐1 was approximately 10‐fold more potent in renal arteries from F‐D/M (EC_50_ = 0.45 ± 0.07 μmol·L^−1^) rats when compared with arteries from F‐P/E rats (EC_50_ = 4 ± 0.3 μmol·L^−1^; Figure 1c). The same oestrous cycle‐dependent differences were observed with mesenteric, coronary and cerebral arteries (Figure 1d–f).

**FIGURE 2:**
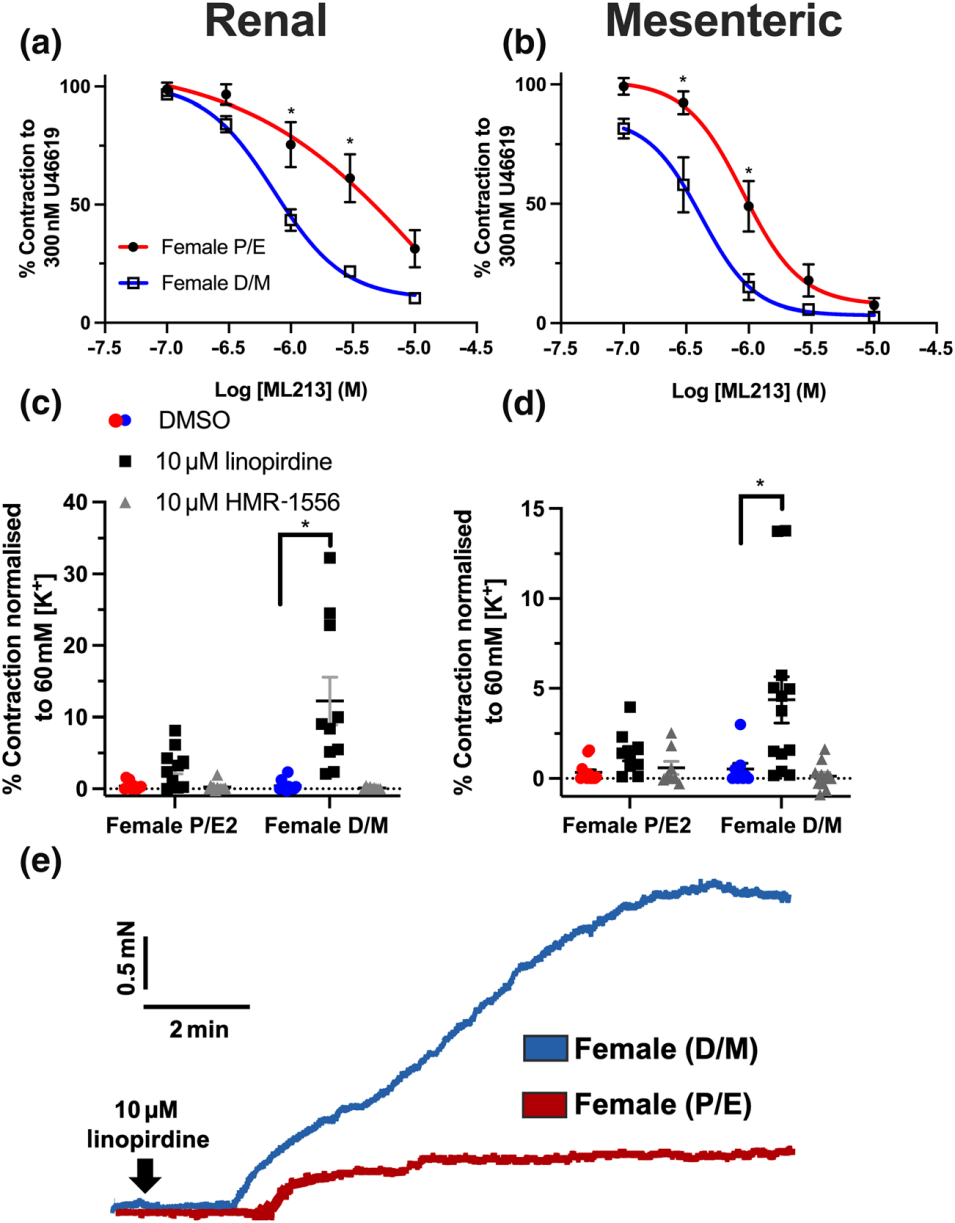
K_V_7 channel modulators are more efficacious and potent on arteries from female D/M, when compared with female P/E Wistar rats. Mean data of relaxation of pre‐contracted tone (U46619; 300 nmol·L^−1^) in response to ML213 (0.01–10 μmol·L^−1^) in renal (n = 5–6; a) and mesenteric (n = 5–6; b) arteries from female pro‐oestrus/oestrus (P/E) and female di‐oestrus/met‐oestrus (D/M) rats. Mean data of increases in basal tone in response to solvent control (DMSO), linopirdine (10 μmol·L^−1^) and HMR‐1556 (10 μmol·L^−1^) in renal (n = 10–12; c) and mesenteric arteries (n = 10–13; d). Representative traces of contraction of renal arteries from female P/E and female D/M rats in response to pan‐K_V_7 channel blocker linopirdine (10 μmol·L^−1^; e)*.* Data shown are means ± SEM; n = number of animals used. *P ≤ 0.05, significantly different from female D/M rats; two‐way ANOVA with post hoc Bonferroni (a, b) or Dunnett (c, d) correction.

The structurally dissimilar K_V_7.2–7.5 activator ML213 (0.1–10 μmol·L^−1^) was also significantly more potent within arteries from F‐D/M rats when compared with arteries from F‐P/E rats (Figure 2a, b). Inhibitors of K_V_7 channels depolarise VSMC membrane potential and produce contraction (Mackie et al., 2008). The pan‐K_V_7 blocker linopirdine (10 μmol·L^−1^) contracted arteries from F‐D/M rats more effectively than arteries from F‐P/E rats (Figure 2c–e). In all groups, no contraction was produced by application of HMR‐1556 (10 μmol·L^−1^), a specific blocker of K_V_7.1 channels (Figure 2c,d), consistent with previous reports (Chadha et al., 2012). These data revealed an oestrous cycle‐dependent contribution of K_V_7.2–7.5 channels to arterial reactivity.

**FIGURE 3:**
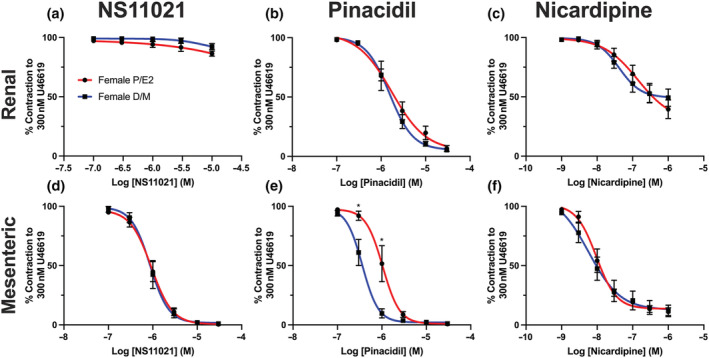
Effect of different ion channel modulators in renal arteries from female (P/E) and female (D/M) Wistar rats. NS11021 (0.1–30 μmol·L^−1^; a, d), pinacidil (0.1–30 μmol·L^−1^; b, e) and nicardipine (0.001–1 μmol·L^−1^; c, f) mediated relaxation of pre‐constricted arterial tone (300 nmol·L^−1^ U46619) in renal (a–c) and mesenteric (d–f) arteries from female pro‐oestrus/oestrus (P/E; red; n = 5) and female di‐oestrus/met‐oestrus (D/M; blue; n = 5–7) Wistar rats. Data shown are means ± SEM; n = number of animals used. * *P ≤* 0.05, significantly different from D/M; two‐way ANOVA with post hoc Bonferroni test.

In contrast, pinacidil‐ and nicardipine‐dependent relaxations of pre‐contracted renal arteries were independent of oestrous cycle stage (Figure 3b,c), whereas NS11021 was ineffective in any stage (Figure 3a). In mesenteric arteries, NS11021 and nicardipine relaxed pre‐contracted tone, independent of oestrous cycle stage (Figure 3d,f). Pinacidil was more potent in mesenteric arteries from F‐D/M rats compared with F‐P/E (Figure 3e; *P* ≤ 0.05). No significant differences were observed in the stable pre‐contracted tone in response to 300 nmol·L^−1^ U46619 (ΔmN; Figure S3) in either mesenteric or renal arteries taken from F‐P/E or F‐D/M Wistar rats.

### 3.2 Diminished K_V_7 channel contribution to receptor‐mediated responses underlies oestrous cycle‐dependent changes in vascular reactivity

**FIGURE 4:**
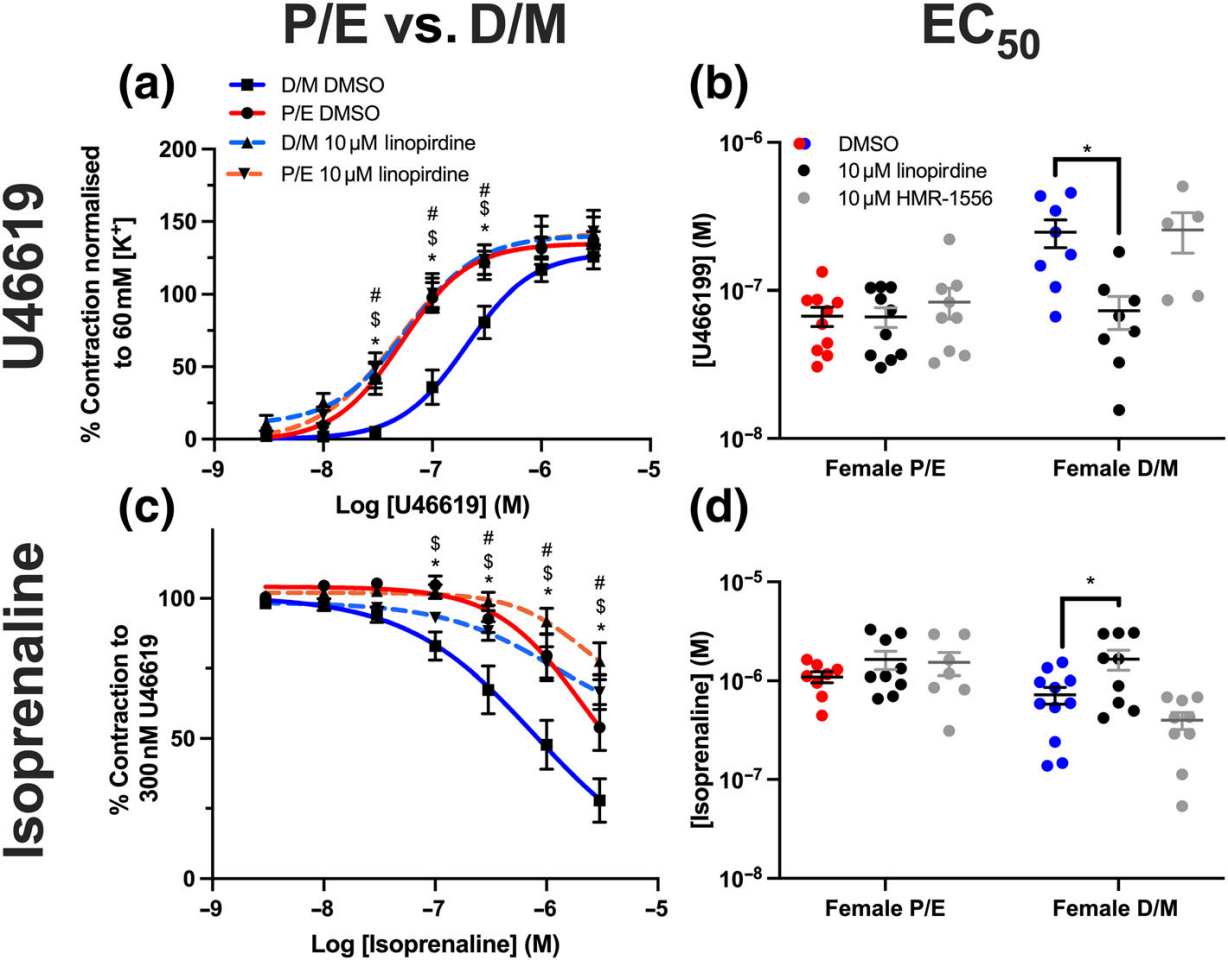
Linopirdine alters receptor‐mediated responses in renal arteries from female D/M, but not female P/E Wistar rats. Mean data of contraction in response to U46619 (0.003–3 μmol·L^−1^; n = 7–10; a) and relaxation of pre‐contracted arterial tone (300 nmol·L^−1^ U46619) in response to isoprenaline (0.003–3 μmol·L^−1^; n = 9–10; c) in renal arteries from F‐D/M and F‐P/E rats, pre‐incubated in DMSO solvent control or linopirdine (10 μmol·L^−1^). Scatter graph representing the raw EC_50_ values of U46619‐mediated contraction (b) or isoprenaline‐mediated relaxation (d) in renal arteries of the mean data to the left, in addition to vessels pre‐incubated in K_V_7.1‐specific blocker HMR‐1556 (10 μmol·L^−1^). Data shown are means ± SEM; n = number of animals used. In (a, c), * *P ≤* 0.05, D/M DMSO significantly different from P/E DMSO; # *P ≤* 0.05, D/M DMSO significantly different from D/M 10 μmol·L^−1^ linopirdine; $ *P ≤* 0.05, D/M DMSO significantly different from P/E 10 μmol·L^−1^ linopirdine; in (b, d), * *P ≤* 0.05, significantly different as indicated; two‐way ANOVA with a post hoc Bonferroni (a, c) or Dunnett (b, d) correction.

K_V_7 channel blockers, such as linopirdine, enhance receptor‐mediated contractions (Brueggemann et al., 2006) and diminish cAMP‐PKA‐dependent β‐adrenoreceptor‐mediated vaso‐relaxation (Chadha et al., 2012). Within this study, contraction mediated by U46619 (0.003–3 μmol·L^−1^) was less potent in renal arteries from F‐D/M compared with F‐P/E rats (Figure 4a,b). Linopirdine significantly augmented the sensitivity of U46619‐mediated vasoconstriction within vessels from F‐D/M, but not F‐P/E rats (Figure 4a,b). In arteries from F‐D/M rats pre‐incubated in linopirdine, the response to U46619 was equipotent to arteries from F‐P/E rats pre‐incubated in both DMSO solvent control and linopirdine (Figure 4b). In contrast, pre‐incubation with linopirdine had no effect in arteries from F‐P/E rats, and HMR‐1556 had no effect within any group (Figure 4a,b).

Isoprenaline was significantly less potent in renal arteries from F‐P/E when compared with vessels from F‐D/M rats (Figure 4c, d). As previously reported (Chadha et al., 2012), linopirdine significantly attenuated isoprenaline‐mediated vasorelaxation in arteries from F‐D/M rats (Figure 4c, d). By contrast, the effect of linopirdine on isoprenaline‐mediated vasorelaxation in arteries from F‐P/E rats was smaller (Figure 4c, d). The response to isoprenaline in arteries from F‐D/M rats pre‐incubated in linopirdine was equipotent to arteries from F‐P/E rats pre‐incubated in either DMSO solvent control or linopirdine (Figure 4d). Moreover, in all vessels, pre‐incubation with HMR‐1556 had no effect (Figure 4d). See Figure S4 for U46619‐mediated contraction and Figure S5 for isoprenaline‐mediated relaxation within mesenteric, cerebral and coronary arteries from F‐P/E and F‐D/M rats. Within these arteries, where linopirdine sensitivity was observed, significant differences in control conditions between F‐D/M and F‐P/E rats were also observed.

The aggregated findings indicate that the K_V_7.2–5 channel contribution to isoprenaline‐ and U46619‐mediated vascular response was diminished within arteries from F‐P/E rats. This finding potentially underlies the observed oestrous cycle‐dependent increased sensitivity to TXA2 receptor‐mediated contraction and decreased sensitivity to β‐adrenoreceptor‐mediated relaxation in arteries from F‐P/E, when compared with F‐D/M rats.

### 3.3 Identification of K_V_7 channel transcript and protein expression in arteries from female Wistar rats

The molecular characteristics of the candidates for vascular K_V_7 function were subsequently determined. RT‐qPCR revealed no significant differences in *Kcnq1–5* nor β‐auxiliary subunit *Kcne1–5* relative transcript abundance within renal and mesenteric arteries from both groups (2^−ΔCq^; Figure S6A–D). Positive control samples (brain and heart) for transcript expression of target genes can also be seen in Figure S5 (Figure S6E–H).

**FIGURE 5:**
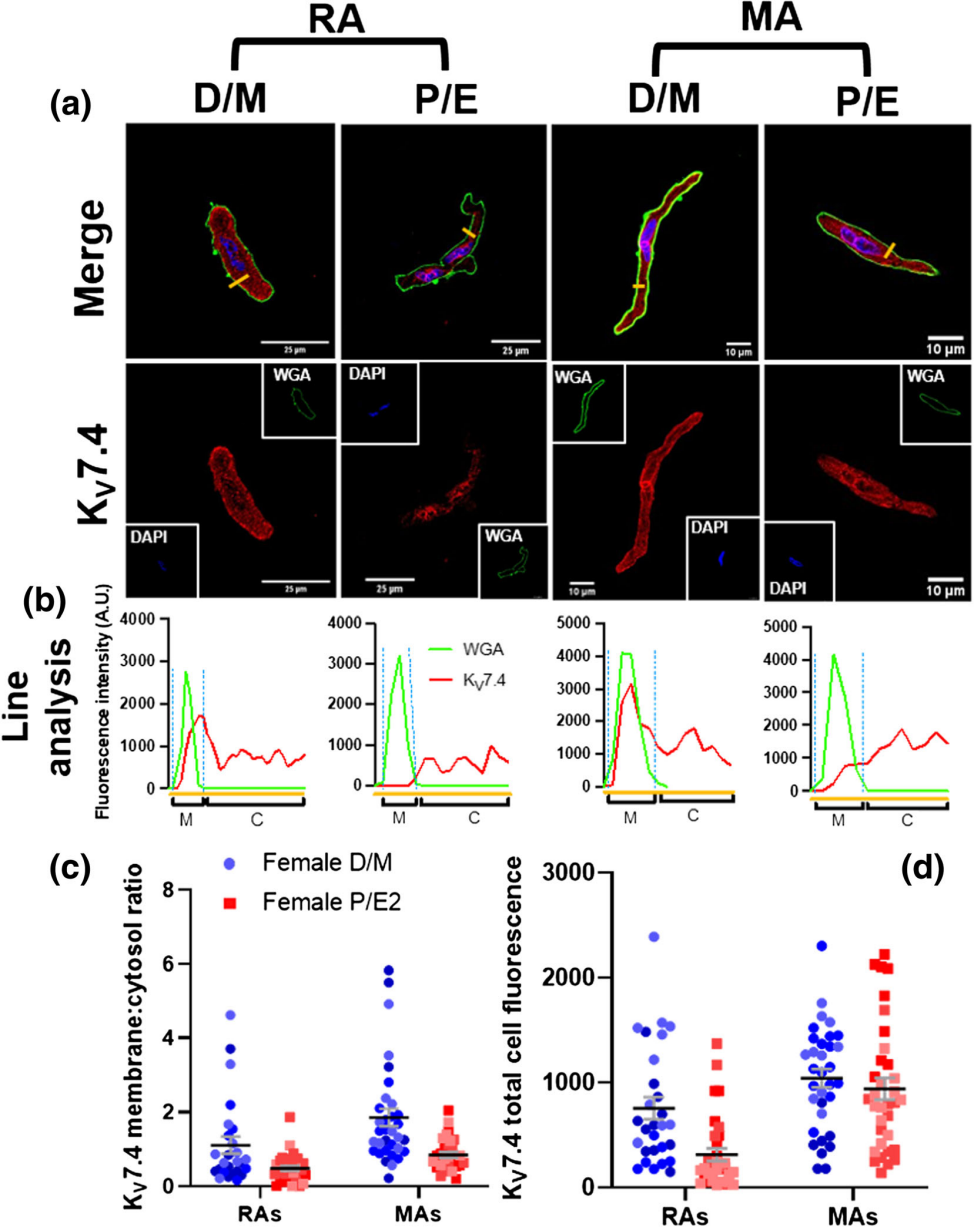
Oestrous cycle‐dependent reduction in K_V_7.4 subcellular distribution in mesenteric and renal artery myocytes. Representativeimages of immunocytochemistry demonstrate K_V_7.4 expression (red) within renal (RA)and mesenteric (MA; a) artery vascularsmooth muscle cells from female di‐oestrus/met‐oestrus (D/M) and female pro‐oestrus/oestrus (P/E) Wistar rats. Plasma membraneand nuclear markers, wheat germ agglutinin (WGA; green) and 4′,6‐diamidino‐2‐phenylindole (DAPI; blue), respectively, are alsoshown. Fluorescence intensity profiles were plotted for K_V_7.4 and WGA measured in arbitrary units (A.U.) along the yellow line seenin the merged image above (b). Fluorescence intensity ≥ 200 A.U. was considered as the plasma membrane (M), and below the threshold was considered as the cytosol (C; b). The membrane:cytosol ratio for K_V_7.4 expression was calculated by measuring thefluorescence intensity of K_V_7.4 within the membrane and dividing it by the fluorescence intensity of K_V_7.4 within cytosol from threerandomly drawn lines*.* Data shown are scatterplots for membrane:cytoplasm ratio (c)and total fluorescence (d)from 8‐12 individualcells isolated from 3rats. Different colours represent different animals. The mean ± SEM are included in theplot.

Immunostaining of K_V_7.4 was performed in VSMCs isolated from renal and mesenteric arteries, with 8‐12 cells imaged per rat. Preliminary data indicated K_V_7.4‐associated fluorescence to be predominantly in the periphery, overlapping with the membrane marker WGA in VSMCs from F‐D/M rats. In contrast, K_V_7.4 staining was diffuse throughout the cytosol of VSMCs from F‐P/E rats (Figure 5a,b). When comparing biological replicates, membrane:cytosol ratio for K_V_7.4 changed from 1.2 ± 0.19 and 1.8 ± 0.2 in renal and mesenteric artery myocytes from F‐D/M rats, respectively, to 0.5 ± 0.03 and 0.8 ± 0.02 in the corresponding cells from F‐P/E rats (Figure 5c). A reduction in total cell fluorescence (A.U.) was observed between renal, but not mesenteric VSMCs between the groups (Figure 5d). Similar experiments failed to reveal comparable oestrous cycle‐dependent differences in staining for the other K_V_7 subtypes associated with vascular function, K_V_7.1 and K_V_7.5, or the β‐auxiliary subunit protein Kcne4 (Figure S7). Therefore, further experiments focused on K_V_7.4 alone. As the number of animals sampled was only 3, we did not apply statistical analysis to these exploratory findings.

### 3.4 Cyclical increases in plasma oestradiol mediate a reduction in K_V_7.4 membrane abundance via GPER1, impairing ML213‐mediated relaxation

To identify potential candidates that drive the observed oestrous cycle‐dependent fluctuation in K_V_7.4 membrane abundance and function, sex steroids were extracted from plasma from animals,obtained immediately after killing. Concentrations of circulating steroidal and gonadal hormones were determined via ELISA and LC–MS/MS (Table 2). Plasma E2 was significantly higher in samples from F‐P/E rats, compared with that found in F‐D/M, whereas levels of progesterone was conversely different (Table 2). No differences between F‐D/M and F‐P/E values were observed with regard to the other hormones assayed (Table 2).

**TABLE 2:** Plasma hormone concentrations

Hormone	F‐P/E			F‐D/M			Student's *t* test
	plasma concentration (ng·ml^−1^)	SEM (±)	n	plasma concentration (ng·ml^−1^)	SEM	n	
Oestradiol	0.36	0.005	8	0.019	0.005	8	*
Testosterone	0.04	0.018	8	0.023	0.005	8	ns
Androstenedione	0.101	0.42	8	0.063	0.015	8	ns
Progesterone	2.977	0.28	6	5.802	1.217	6	*
Aldosterone	0.018	0.006	8	0.017	0.004	8	ns
Follicular stimulating hormone	0.972	0.174	14	0.958	0.274	14	ns
Luteinising hormone	3.499	0.655	14	3.373	0.368	14	ns

*Note*: Hormonal plasma concentration was determined using liquid chromatography–tandem mass spectrometry (steroids) and enzyme‐linked immunoblot assay (ELISA) (luteinising hormone and follicular stimulating hormone) and expressed as ng·ml^−1^ in female rats during either pro‐oestrus/oestrus (F‐P/E) or di‐oestrus/met‐oestrus (F‐D/M). Data shown are means, with SEM and number of animals used (n).

* *P* ≤ 0.05, significant difference between F‐P/E and F‐D/M groups; ns, no significant difference; Student's *t* test.

The onset of a pro‐contractile phenotype in arteries from F‐P/E occurs within a narrow time frame in the absence of a change in the relative abundance of *Kcnq*/*Kcne* transcripts. In addition, E2‐mediated internalisation of K_V_7.1 channels occurred via a fast‐acting, ‘non‐genomic’ signalling cascade (Alzamora et al., 2011; Rapetti‐Mauss et al., 2013). We proposed that a rise in plasma E2 during pro‐oestrus mediates a reduction in K_V_7.4 membrane abundance in a ‘non‐genomic’ process, similar to previous reports (Rapetti‐Mauss et al., 2013), which does not recover until met‐oestrus. The three principal E2 receptors comprise ERα and ERβ, canonically considered nuclear receptors, and a novel membrane bound receptor, GPER1, that are encoded for by *Ers1*, *Ers2* and *Gper1*, respectively. We ascertained the expression of these receptors in rat arteries using whole uterine lysates as a positive control. Rat mesenteric and renal artery lysates from both groups had an expression profile of *Esr1* > *Gper1* > *Esr2*, whereas the expression profile in uterus was *Esr1* > *Esr2 > Gper1* (Figure S8A–C).

We then determined whether short‐term treatment with exogenous E2 could mimic the oestrous cycle‐dependent changes in K_V_7 responses. Renal and mesenteric arteries from female Wistar rats were incubated with E2 for a period of 5 or 30 min. In mesenteric and renal arteries from F‐D/M rats, 5 min and 30 min of E2 pre‐incubation significantly impaired ML213‐mediated relaxation (Figure S9A,C). Comparable treatment with exogenous E2 had no effect in arteries from F‐P/E animals (Figure S9B,D). Additional experiments were undertaken to determine the long‐term and potential genomic effects of incubation with exogenous E2 on ML213‐mediated relaxation of pre‐contracted mesenteric arteries. 4 h incubation with E2, and vessels that were incubated with E2 for 10 min, then washed and left for 4 h prior to application of ML213 were compared against vessels pre‐incubated in solvent control (Figure S9E,F). In both conditions, E2 attenuated ML213‐mediated relaxation only in arteries taken from F‐D/M rats (Figure S9E,F).

**FIGURE 6:**
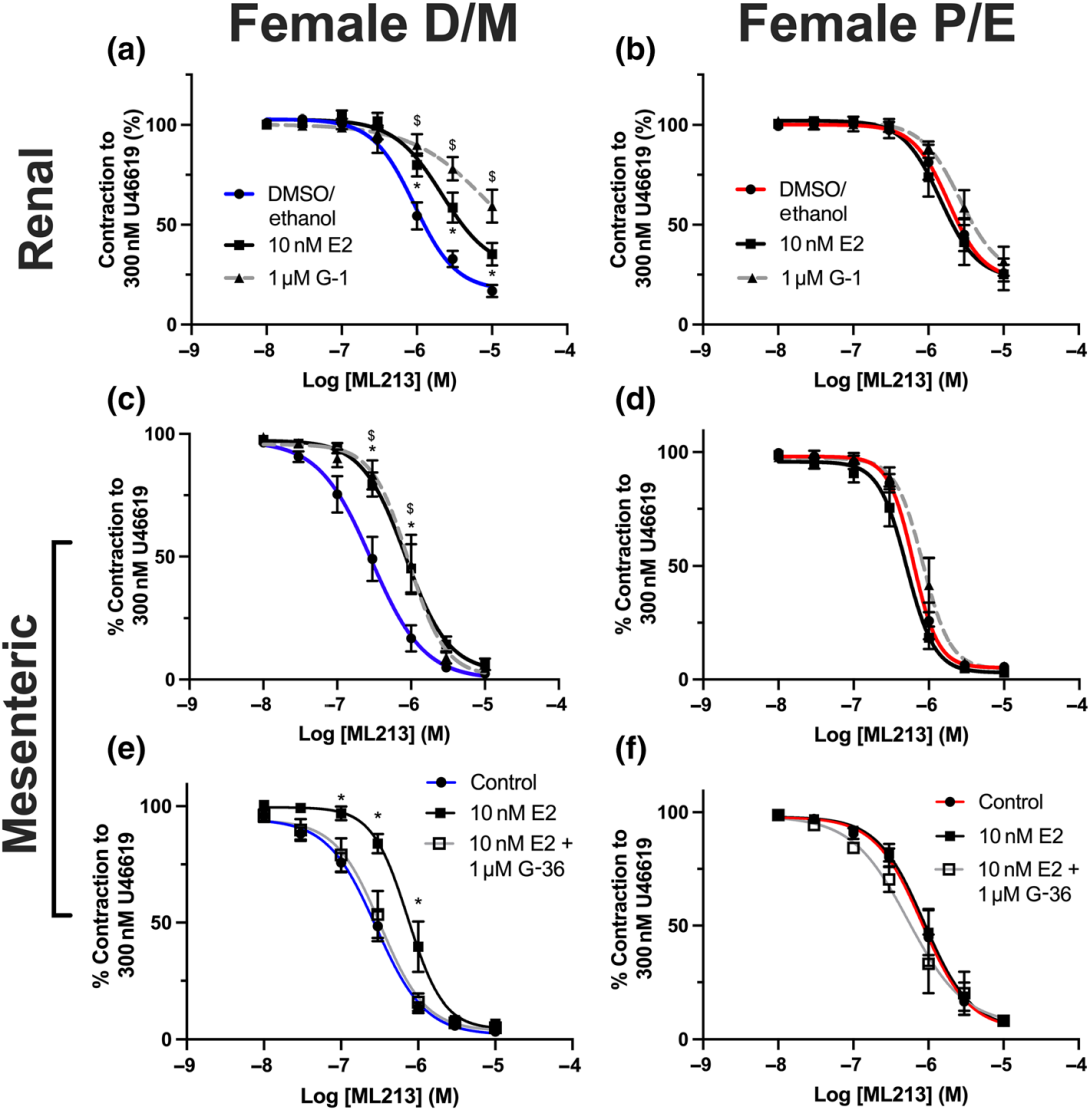
Oestradiol attenuation of the effects of ML213 is GPER1‐mediated. Mean data for ML213‐mediated relaxation of pre‐constricted arterial tone (U46619; 300 nmol·L^−1^) in renal (a, b) and mesenteric (c–f) arteries from female Wistar rats in di‐oestrus/met‐oestrus (F‐D/M; a–c; n = 5–12) or pro‐oestrus/oestrus (F‐P/E; d–f; n = 6–11) pre‐incubated in the DMSO/ethanol solvent control (F‐D/M; F‐P/E), oestradiol (E2; 10 nmol·L^−1^) or GPER1 agonist G‐1 (1 μmol·L^−1^, a–d) or GPER1 antagonist G‐36 (1 μmol·L^−1^) in combination with E2 (10 nmol·L^−1^; e, f). Data shown are means ± SEM; n = number of animals used. */$ *P ≤* 0.05, significantly different from solvent control; two‐way ANOVA with post hoc Dunnett's test.

Pre‐incubating renal and mesenteric arteries from F‐D/M rats with the specific GPER1 agonist G‐1 (1 μmol·L^−1^) attenuated ML213‐mediated relaxations in a fashion analogous to pre‐incubation with E2 (Figure 6a,c). Again, G‐1 pre‐incubation had no effect on renal (Figure 6b) or mesenteric (Figure 6d) arteries from F‐P/E rats. The response to ML213 in mesenteric arteries from F‐D/M pre‐incubated in E2 (EC_50_ = 1 ± 0.17 μmol·L^−1^) or G‐1 (EC_50_ = 0.9 ± 0.17 μmol·L^−1^) mirrored the profile for ML213 seen in mesenteric arteries from F‐P/E Wistars pre‐incubated in solvent controls (EC_50_ = 0.84 ± 0.1 μmol·L^−1^; Figure 6c,d). Moreover, pre‐incubating mesenteric arteries with the selective GPER1 antagonist G‐36 (1 μmol·L^−1^; Dennis et al., 2011) prior to application of E2 prevented its inhibitory effects on ML213‐mediated relaxation (Figure 6e). In contrast, G‐36 had no effect on ML213‐mediated relaxation in arteries from F‐P/E rats (Figure 6f).

**FIGURE 7:**
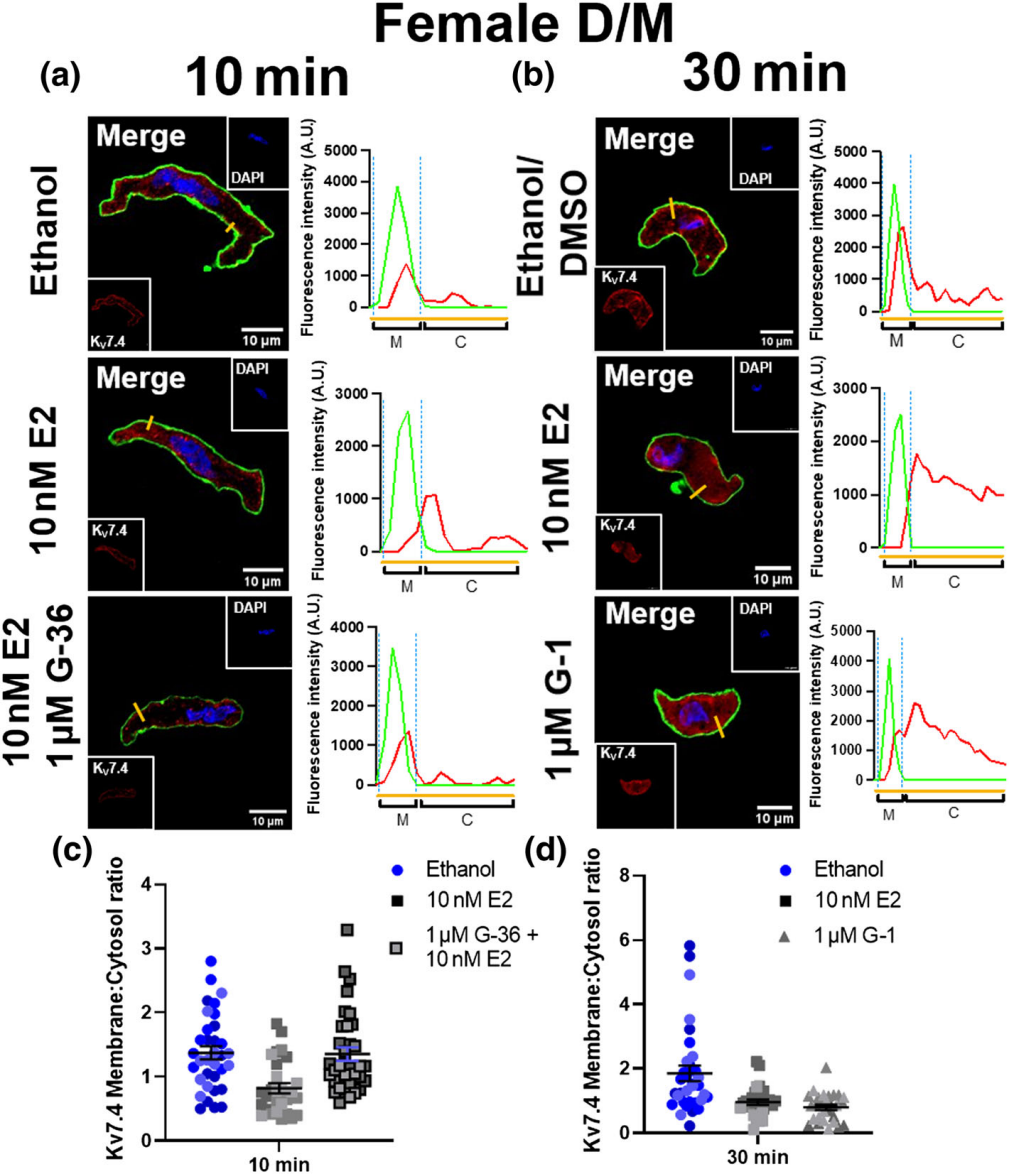
Oestradiol E2 incubation diminishes K_V_7.4 membrane abundance in isolated mesenteric artery vascular smooth muscle cells from female D/M Wistar rats. Representative images of immunocytochemistry demonstrate K_V_7.4 expression (red) in mesenteric artery vascular smooth muscle cells from females in di‐oestrus/met‐oestrus (D/M), pre‐incubated in either solvent control (ethanol/ DMSO), oestradiol (E2; 10 nmol·L^−1^) or E2 + GPER1 antagonist G‐36 (1 μmol·L^−1^) for 10 min (a) or ethanol/DMSO, E2 (10 nmol·L^−1^) or GPER1 agonist G‐1 (1 μmol·L^−1^) for 30 min (b). Plasma membrane and nuclear markers wheat germ agglutinin (WGA; green) and 4′,6‐diamidino‐2‐phenylindole (DAPI; blue) are also shown. Fluorescence intensity profiles were plotted for K_V_7.4 and WGA measured in arbitrary units (A.U.) along the yellow line seen in the merged image above. Fluorescence intensity ≥ 200 A.U. was considered the plasma membrane (M), and below the threshold was considered the cytosol (C). The membrane:cytosol ratio for K_V_7.4 expression was calculated by measuring the fluorescence intensity of K_V_7.4 within the membrane and dividing it by the fluorescence intensity of K_V_7.4 within cytosol from three randomly drawn lines in individual cells. Data shown are scatterplots for membrane:cytoplasm ratio (c,d) from 10‐12 individual cells isolated from 3 rats. Different colours represent different animals. The mean ± SEM are included in the plot.

In a subsequent set of exploratory experiments, isolated mesenteric artery VSMCs from F‐DM rats were incubated with either E2 (10 nmol·L^−1^) for 10 min (Figure 7a) or 30 min (Figure 7b). The data suggests an E2‐mediated reduction in the overlap of K_V_7.4 staining with WGA when compared with the corresponding solvent controls (Figure 7a,b), and a reduction in the membrane:cytosol ratio for K_V_7.4 staining (Figure 7b,d), that was analogous to the reduction observed when comparing myocytes from F‐D/M and F‐P/E (Figure 5). Preliminary data also indicated that pre‐incubating isolated VSMCs with the specific GPER1 antagonist G‐36 for 10 min prior to the application for E2 prevented a reduction in K_V_7.4 membrane abundance (Figure 7c), whereas the GPER1 agonist G‐1 replicated E2‐mediated K_V_7.4 translocation (Figure 7d). Further, that neither E2 nor G‐1 had any effect on the predominantly cytosolic staining for K_V_7.4 in VSMCs from mesenteric arteries from F‐P/E Wistar rats (Figure S10A–C). In summary, raised plasma E2 during the P/E stages of the oestrous cycle correlated with, 1.) a potentially diminished K_V_7.4 membrane abundance, and 2.) impaired K_V_7.4 function. Both of these effects were reproduced in arteries from F‐D/M rats by activation of GPER1.

E2 and G‐1 incubation also impaired ML213‐mediated relaxations in mesenteric arteries from male rats, whereas relaxations to the BK_Ca_ activator NS11021 were unaffected by E2 pre‐incubation (Figure S11).

Compared with E2, little is known of the effect of progesterone on K_V_7 channel function. However, as progesterone was significantly raised in the plasma from F‐D/M rats, we assayed the effects of pre‐incubating mesenteric arteries from F‐P/E and F‐D/M rats in progesterone (10 nmol·L^−1^) for 5 and 30 min on ML213‐mediated responses (Figure S12). No change in ML213‐mediated relaxation was observed in any vessels from either group (Figure S12). Consequently, progesterone was not considered in the following investigations but was helpful in confirming cycle stage.

### 3.5 Oestrogenic inhibition of K_V_7 activator‐mediated relaxation is not endothelium‐dependent

**FIGURE 8:**
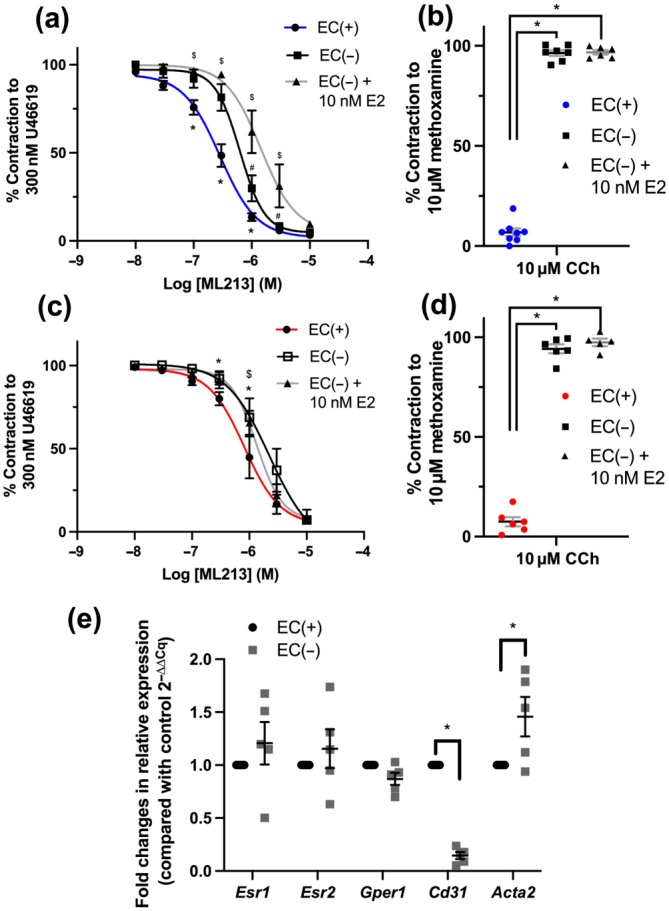
Attenuation by oestradiol of ML213‐mediated relaxation is not endothelium dependent. Mean data for ML213‐mediated relaxation of pre‐constricted arterial tone (U46619; 300 nmol·L^−1^) in arteries from female Wistar rats in di‐oestrus/met‐oestrus (F‐D/M; a, b; n = 6–8) or pro‐oestrus/oestrus (F‐P/E; c, d; n = 5–6) in the presence and absence of endothelial cells (ECs(+)/(−)) and in the absence of endothelial cells pre‐incubated in oestradiol (E2, 10 nmol·L^−1^; EC(−) + E2). Mean data and scatter plot for carbachol (CCh)‐mediated relaxation of pre‐contracted arterial tone (10 μmol·L^−1^ methoxamine) generated within the same vessels prior to application of ML213 (b, d). Relative fold expression in oestrogen receptors (*Esr1*, *Esr2* and *Gper1*), EC marker *Cd31* and vascular smooth muscle marker *Acta2* in whole lysates of mesenteric arteries from female Wistar rats in vessels denuded of endothelium (EC(−)) compared with vessels with intact endothelium (EC(+); 2^−ΔΔCq^; n = 5; e). Data shown are means ± SEM; n = number of animals used. * *P ≤* 0.05, significantly different from EC(+); $ *P ≤* 0.05, significant difference between EC(+) and EC(−) + 10 nM E2; # *P ≤* 0.05, significant difference between EC(−) and EC(−) + 10 nM E2: two‐way ANOVA with post hoc Bonferroni test.

A series of experiments were performed to ascertain if the modulatory effects of GPER1 activation were dependent on the presence of functional endothelium. Similar to our previous findings, removing the endothelium significantly attenuated ML213‐mediated relaxation in mesenteric arteries from both F‐D/M and F‐P/E rats (Figure 8a,c). Pre‐incubating EC‐denuded vessels from F‐D/M rats with E2 (10 nmol·L^−1^) additively attenuated ML213‐elicited relaxation (Figure 8a), whereas E2 pre‐incubation had no effect in EC‐denuded arteries from F‐P/E rats (Figure 8c; n = 5–6). The relative fold change in expression of oestrogen receptor and VSMC and EC marker transcripts was compared between EC(+) and EC(−) vessels, using whole lysates (2^−ΔΔCq^). When comparing relative transcript abundance between EC(−) and EC(+) arteries, a significant reduction in *Cd31* (platelet or endothelial cell marker 1) transcript was observed in conjunction with a significant increase in that of *Acta2* (α‐smooth muscle actin; Figure 8e). A small increase in *Ers1* and *Ers2* transcripts and a minor decrease in *Gper1* transcripts were observed in EC(−) lysates when compared with EC(+) lysates (Figure 8e), although this failed to reach significance. In summary, although GPER1 expression was moderately higher within the endothelium, the effect of GPER1 signalling on vascular K_V_7.4 appears to originate from within the smooth muscle.

### 3.6 GPER1 activation reduced K_V_7.4 currents

**FIGURE 9:**
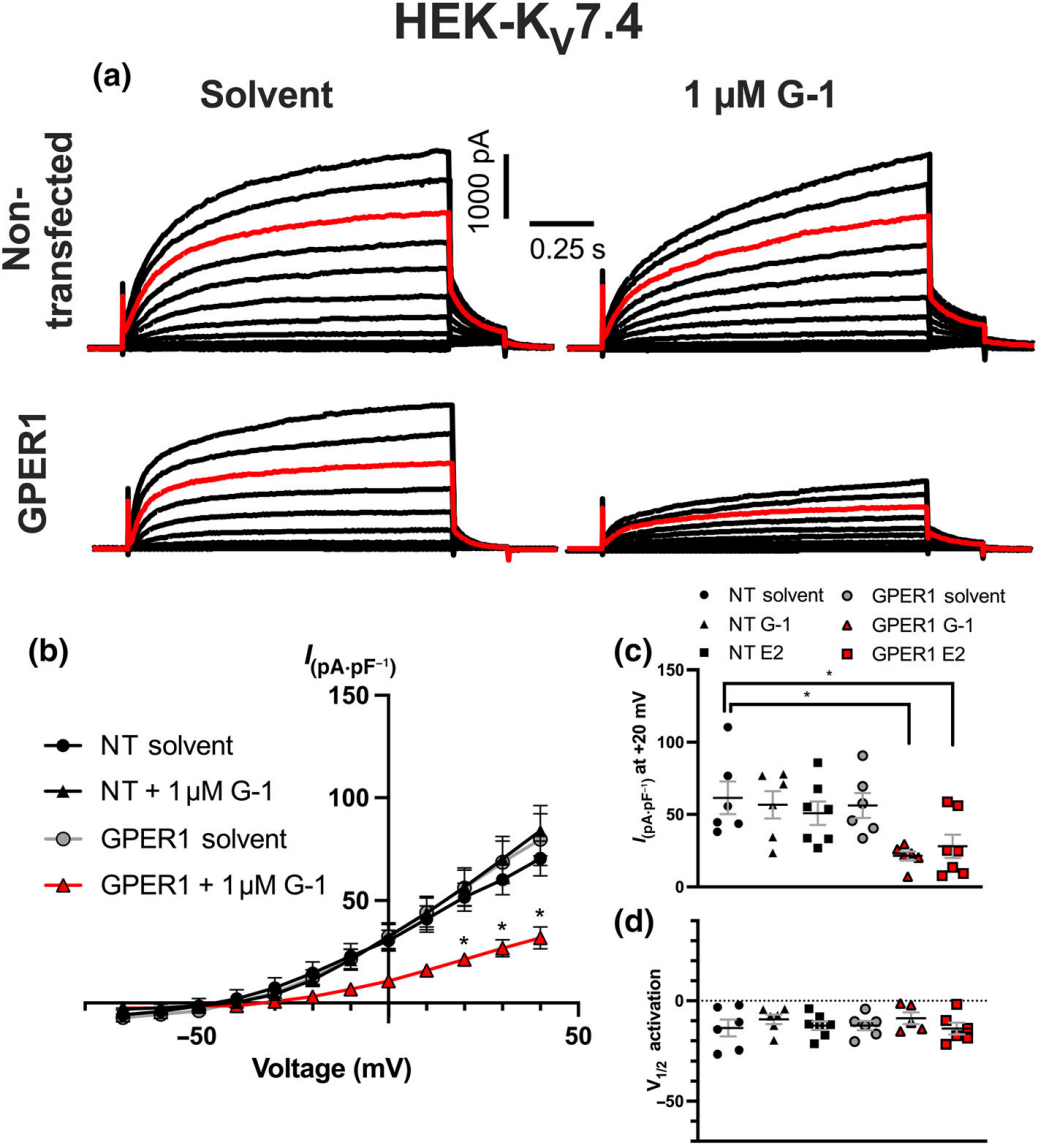
Pre‐incubation with oestradiol (E2) or G‐1 impairs K_V_7.4 channel activity in GPER1‐transfected cells. Representative current recordings during ruptured whole‐cell voltage clamp in non‐transfected (NT) HEK‐K_V_7.4 cells, pre‐incubated (30 min) in solvent control (upper left) or G‐1 (1 μmol·L^−1^; upper right) and GPER1‐transfected cells pre‐incubated in solvent control (lower left) or G‐1 (lower right; a). Red line represents current flow in response to +20 mV. Cells were held at a holding potential of −60 mV and then stepped to test voltage for 1.5 s every 15 s ranging from −70 to +40 mV increasing in 10 mV intervals. Before returning to rest potential, voltage was stepped to an inactivation potential of −40 mV. Mean *IV* relationships plotted for HEK‐K_V_7.4 cells; NT in solvent control (n *=* 5) or G‐1 (n *=* 6) and GPER1‐transfected cells in solvent control (DMSO; n = 6) or G‐1 (n = 6; b). Scatter graph demonstrates peak current amplitude at +20 mV in non‐transfected cells pre‐incubated in solvent control (n = 6), G‐1 (n = 6), oestradiol (E2, 10 nmol·L^−1^, n = 7) and GPER1‐transfected cells solvent control (n = 6), G‐1 (n = 6) or E2 (n = 7; c). Voltage dependence of activation for K_V_7.4 currents (d). Data shown are means ± SEM; n = number of animals used. * *P ≤* 0.05, significantly different as indicated; (b) two‐way ANOVA with post hoc Dunnett's correction, (c, d) one‐way ANOVA.

Ruptured whole‐cell recording from HEK cells expressing K_V_7.4, was used as a secondary means of determining the effect of GPER1 activation on K_V_7.4 channel activity. Incubation of HEK‐K_V_7.4 cells with the GPER1 agonist G‐1 (1 μmol·L^−1^) or E2 (10 nmol·L^−1^) for 30 min produced a considerable reduction in K_V_7.4 currents only in cells expressing GPER1 receptor (Figure 9). GPER1 stimulation by either G‐1 or E2 did not affect voltage of half activation (V_1/2_) of K_V_7.4 currents (Figure 9d). Currents recorded under solvent control conditions were identical in untransfected and GPER1‐expressing HEK cells (Figure 9).

### 3.7 E2 reduces K_V_7.4 interaction with forward trafficking molecular chaperone protein HSP90 in F‐D/M, but not F‐P/E VSMCs

**FIGURE 10:**
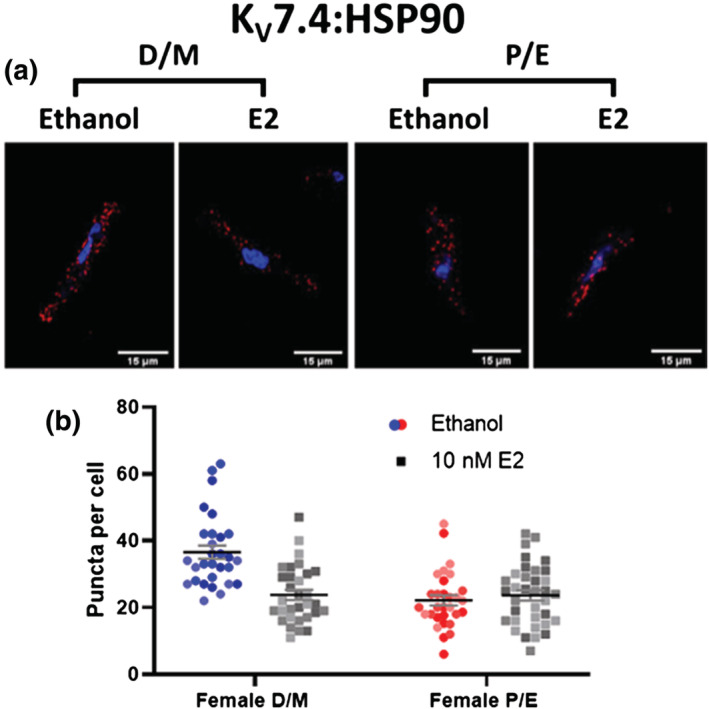
Oestradiol reduces K_V_7.4–heat shock protein 90 interactions in mesenteric artery myocytes from F‐D/M, but not F‐P/E rats. Proximity ligation assay (PLA) for K_V_7.4 and heat shock protein 90 (HSP90) interaction within female di‐oestrus/met‐oestrus (D/M) and female pro‐oestrus/oestrus (P/E) mesenteric artery myocytes pre‐incubated in either solvent control(ethanol) or 10 nmol·L^−1^ oestradiol (E2; 30 min). Representative images of mesenteric artery myocyte mid‐cell cross section exhibit fluorescent puncta that illustrate K_V_7.4:HSP90 interactions within a distance of <40 nm (red) and the nucleoli via 4′,6‐diamidino‐2‐phenylindole (DAPI; blue; a). Data shown are scatterplots for number of puncta per cell (b) from 10‐15 individual cells isolated from 3 rats. Different colours represent different animals. The mean ± SEM are included in the plot.

The molecular chaperone protein HSP90 is critical in the folding and biogenesis of potassium channels including K_ATP_ (Yan et al., 2010), K_V_11.1 (Ficker, 2003) and K_V_7.4 (Gao et al., 2013). Additionally, GPER1 activation increases human myometrial contractility by phosphorylation of HSP27 (Maiti et al., 2011), and infusion of angiotensin II decreased the interaction of K_V_7.4 and HSP90, diminishing K_V_7.4 membrane abundance (Barrese, Stott, Figueiredo, et al., 2018). Therefore, we proposed that the reduction in membrane:cytosol ratio observed in response to E2/G‐1 was mediated by a reduction in the interaction of K_V_7.4 and HSP90, in a process similar to that following infusion of angiotensin II. PLA was used to resolve protein–protein interactions ≤ 40 nm, which are expressed as red puncta within the cell. As the data were derived from less than 5 animals we cannot apply statistical analysis to our findings but the indication is that pre‐incubation for 30 min with E2 (10 nmol·L^−1^) reduced the interaction between K_V_7.4 and:HSP90, within mesenteric VSMCs from F‐D/M Wistar rats, compared with the solvent control (Figure 10). No change in K_V_7.4:HSP90 interactions was observed in similar experiments with VSMCs from F‐P/E rats incubated with E2 (Figure 10b). Additionally, the puncta per cell in VSMCs from F‐P/E rats was equivalent to that observed in VSMCs from F‐D/M pre‐incubated in E2.

## 4 DISCUSSION

In this study, we demonstrate clear oestrous cycle‐dependent changes in vascular reactivity, whereby pro‐contractile vessels from F‐P/E rats exhibited diminished K_V_7.4 channel function and membrane abundance, in conjunction with significantly raised plasma E2. Moreover, a F‐P/E pro‐contractile phenotype could be replicated in pro‐relaxant F‐D/M rats by exposure to E2 or the novel GPER1 activator G‐1, a change that was prevented by the GPER1‐specific inhibitor G‐36. In a heterologous overexpression system, both E2 and G‐1 diminished human K_V_7.4 channel currents only in cells transfected with *GPER1*, independent of a change in the biophysical properties of the current and consistent with a reduction in channel number. Finally, pilot data indicated that exogenous E2 diminished the interaction of K_V_7.4 with its forward trafficking molecular chaperone protein HSP90 only in those rats in a ‘low plasma E2’ stage of the oestrous cycle.

### 4.1 Cyclical reduction in K_V_7.4 membrane abundance correlates with a pro‐contractile phenotype

In spite of the known sexual dimorphisms in cardiovascular physiology and pathophysiology (Pabbidi et al., 2018), little is known about K_V_7 channel activity in arteries from females. Of the few studies to consider sex as a factor, K_V_7 channels within the female were shown to (1) be differentially regulated by its β‐auxiliary subunit protein Kcne4 (Abbott & Jepps, 2016) and (2) to impair noradrenaline‐induced increases in total peripheral resistance in normotensive and spontaneously hypertensive female rats only (Berg, 2018).

Here, we demonstrate that two K_V_7.2–5 channel activators, S‐1 and ML213, relaxed pre‐contracted arterial tone in a range of arteries and the pan‐K_V_7 channel inhibitor linopirdine and not the specific inhibitor of K_V_7.1 channels, HMR‐1556, increased basal tone (Chadha et al., 2012; Mackie et al., 2008; Ng et al., 2011), though significantly more potently and efficaciously in arteries from F‐D/M rats. Additionally, K_V_7.2–5 channel inhibition enhanced TXA_2_‐mediated contractions and impaired β‐adrenoceptor‐driven relaxations, though more effectively in arteries from F‐D/M rats. Immunocytochemistry revealed a corona‐like staining for K_V_7.4 in myocytes from F‐D/M renal and mesenteric arteries that was absent in myocytes from these arteries from F‐P/E rats. However, neither total cell fluorescence nor the relative abundance of the *Kcnq4* transcripts were altered. Thus, our findings indicate a post‐transcriptional cycle‐based reduction in K_V_7.4 membrane abundance, which correlates with diminished contribution of K_V_7 channels to both basal tone and receptor‐mediated responses, contributing to a pro‐contractile phenotype. No change in transcript no membrane abundance of the other candidates for vascular K_V_7 channel function (K_V_7.1, K_V_7.5 and KCNE4) was observed, reinforcing the previous suggestioin that K_V_7.4 is the critical component of the functional K_V_7.4/K_V_7.5 heterotetramer (Barrese, Stott, & Greenwood, 2018). However, other ion channels may also be modulated, as the present study showed significant oestrous cycle‐dependent differences in relaxations to the K_ATP_ activator pinacidil in mesenteric arteries. Further research will focus on this aspect.

### 4.2 E2 may diminish K_V_7.4 membrane abundance through reduced interaction with HSP90, via GPER1 signalling

When screening for candidates that drive the oestrous cycle‐dependent differences in vascular K_V_7 activity, our data revealed an increase in plasma E2 in F‐P/E rats. The role for E2 in modulating vascular reactivity is complex. E2 up‐regulates the bioavailability of nitric oxide and PGI_2_ within ECs and decreases intracellular calcium availability in VSMCs (Mazzuca et al., 2015; Novella et al., 2019). However, there is less consensus in the published reports of ion channel modulation by E2. E2 is known to both increase and decrease the activity of ion channels such as BK_Ca_, K_ATP_ and K_V_ (Kow & Pfaff, 2016). With regard to K_V_7 channels, E2 rapidly internalises K_V_7.1 in crypt cells of the distal colon in female rats, in a fast‐acting, non‐genomic signalling cascade (O'Mahony et al., 2007; Rapetti‐Mauss et al., 2013). E2 also diminished *I*_Ks_ currents in overexpression models and rabbit cardiac myocytes (Busch et al., 1996; Möller & Netzer, 2006) but increased M‐currents (K_V_7.2/3) in mouse NPY neurons (Roepke et al., 2011). Very little is known of the effect of E2 on vascular K_V_7 channels. E2 injection into rats with bilateral ovariectomy significantly increased mean arterial pressure (Takezawa et al., 1994). Here, short incubation with supplemental E2 reduced the extent of ML213‐mediated relaxation in arteries from F‐D/M rats, in an endothelium‐independent process. No additive inhibition of function by E2 was observed in F‐P/E rats where plasma E2 was higher, supporting a role for oestrogenic signalling in driving the observed cycle‐dependent shifts in vasoreactivity. Our exploratory evidence indicated that K_V_7.4 membrane abundance and forward trafficking were impaired by exogenous E2, similarly, no effect was observed in F‐P/E rats.

As the onset of a pro‐contractile phenotype during the oestrous cycle occurs within a narrow time frame, we postulated that the effects of oestrogenic signalling were also non‐genomic. The candidates for fast‐acting oestrogenic signalling include the membrane‐associated GPER1 (Filardo et al., 2000) and ERα following palmitoylation at Cys446 (Simoncini et al., 2000). As mentioned above, E2 rapidly reduced colonic crypt cell conductance via ERα down‐regulation of K_V_7.1 channels via fast‐acting processes that were PKA–PKCδ dependent (O'Mahony et al., 2007, 2009). Here, the effects of extraneous E2 on K_V_7.4 function and membrane abundance were replicated by a specific agonist of GPER1, G‐1 (Bologa et al., 2006) and prevented by the GPER1 antagonist G‐36 (Dennis et al., 2011), indicating a role for GPER1 rather than ERα/β. This was supported by single‐cell electrophysiology in a heterologous overexpression system, whereby E2/G‐1 regulation of K_V_7.4 channels was dependent on GPER1 expression. GPER1 activation mediated a reduction in total K_V_7.4 current, independent of a change in the conductance of the individual channel, further supporting a role for GPER1. However, as longer‐term (4 h) incubation with E2 also inhibited ML213‐mediated relaxation in vessels from F‐D/M rats, we cannot rule out a contribution from the nuclear oestrogen receptors ERα/β. Further, although aldosterone cannot bind GPER1 (Cheng et al., 2014), aldosterone mediates GPER1‐dependent sensitisation to angiotensin II (Batenburg et al., 2012) and phenylephrine‐mediated contractions (Ferreira et al., 2015). Current understanding indicates that aldosterone‐mediated, GPER1‐sensitive, vascular effects may be derived from cross talk between mineralocorticoid and oestrogen receptors (Barton & Meyer, 2015). Although we observed no differences in plasma aldosterone levels between F‐D/M and F‐P/E females, further studies aiming to characterise GPER1 signalling should consider receptor cross talk.

Our data suggest that GPER1 activation alters the forward trafficking of K_V_7.4 through altered interaction with the chaperone HSP90. Angiotensin II also alters HSP90:K_V_7.4 association, resulting in channel ubiquitination and proteasomal degradation (Barrese, Stott, Figueiredo, et al., 2018). We do not know whether similar signalling occurs during the oestrous cycle and channel protein is created de novo or ultimate degradation is prevented and the existing K_V_7.4 can recycle back to the membrane, as shown previously (Rapetti‐Mauss et al., 2013). Moreover, we do not know the signals linking GPER1 activation to HSP90 instability. As there is growing appreciation for the importance of ion channel membrane trafficking as the basis for many channelopathies (Curran & Mohler, 2015), the mechanisms linking GPER1 to HSP90 should be the focus of further studies.

### 4.3 Perspectives

Diminished K_V_7.4 channel function in response to increased plasma E2 has considerable implications for women's health. Mean arterial pressure is reportedly higher in the luteal phase of the menstrual cycle (Danborno et al., 2018), a phase historically associated with progesterone production from the corpus luteum. However, Stricker et al. (2006) demonstrated that E2 levels within mid‐luteal phase were greater than in the early follicular phase. Further, hormone replacement therapy (HRT) has become one of the most controversial topics of women's health of the last three decades. Trends in disease outcomes for patients on combined oestrogen/progestin in the Heart and Estrogen/progestin Replacement Study (HERS) I (Hulley et al., 1998) and II (Hulley et al., 2002) were not favourable, as adverse cardiovascular events were increased. However, there is conflict within the literature (Yang & Reckelhoff, 2011), as animal and human studies on HRT, prior to the HERS, had positive outcomes. Although the effect of E2 on the prevalence of cardiovascular disease in humans remains unclear, an extrapolation of the findings detailed here could imply diminished K_V_7 channel function in the detrimental attributes of exogenous E2 in rodents and humans, as a reduced K_V_7 channel membrane abundance is associated with the hypertensive phenotype. Additionally, aldosterone meditates increased vascular resistance and an increase in blood pressure. Interaction between the mineralocorticoid receptor and GPER1 may diminish K_V_7 function, contributing to aldosterone‐mediated changes in blood pressure. Although GPER1 is largely viewed as a promising therapeutic target in the treatment of cardiovascular disease, we would argue that its effects are currently incompletely understood, meriting further investigation.

## AUTHOR CONTRIBUTIONS

SNB and EAF performed the functional and molecular research. NZMH and RA executed and interpreted the steroid assays. BEI provided essential tools and reagents. SNB and IAG wrote the manuscript. SNB, VB, JBS and IAG designed the research study. All authors contributed to the manuscript and approved the submitted version.

## CONFLICT OF INTEREST

The authors declare no conflict of interest.

## DECLARATION OF TRANSPARENCY AND SCIENTIFIC RIGOUR

This Declaration acknowledges that this paper adheres to the principles for transparent reporting and scientific rigour of preclinical research as stated in the *BJP* guidelines for Design and Analysis, Immunoblotting and Immunochemistry, and Animal Experimentation, and as recommended by funding agencies, publishers and other organisations engaged with supporting research.

## Supporting information


**Figure S1:** Cervical histology throughout the oestrus cycle.
Representative images of cell suspension lifted from the cervix during met‐oestrus (Ai/Aii), di‐oestrus (Bi/Bii), pro‐oestrus (Ci/Cii) and oestrus (Di/Dii) at magnifications x10 (Ai‐Di), x20 (Aii,Cii,Dii) and x40 (Bii) and stained in toluidine blue. Arrows demonstrate nucleated epithelial cells (orange), anucleated epithelial cells (blue) and leukocytes (green; Aii‐Dii). Grey arrow demonstrates a ‘swirl’ of nucleated epithelial cells typical of pro‐oestrus, used as an additive tool in determining cycle stage (Cii).
**Figure S2:** Validating Anti‐K_V_7.4 #APC‐164.
Chinese hamster ovarian (CHO) cells transfected with plasmids containing *Kcnq4* presented with diffuse labelling for K_V_7.4 (A; red). Comparably, no staining was observed in *Kcnq4‐*transfected CHO cells supplemented with blocking peptide (#BLP‐PC164; B) nor non‐transfected CHO cells (C). Similarly, male mesenteric artery (MA) vascular smooth muscle cells presented with diffuse labelling for K_V_7.4 (D), but not when supplemented with blocking peptide (#BLP‐PC164; E) nor in in the absence of a primary antibody (Noº Ab control; F). 4′,6‐diamidino‐2‐phenylindole (DAPI; blue; A‐F), wheat germ agglutinin membrane marker (green) merged image (merge; D‐F) insets.
**Figure S3:** Pre‐contracted arterial tone in arteries from female rats prior to application of ion channel modulators.
Mean data and scatter plot for stable ΔmN in response to 300 nmol‐L^−1^ U46619 in renal (A) and mesenteric (B) arteries taken from females in Di‐oestrus (F‐D/M; blue) and females in Pro‐oestrus and Oestrus (F‐P/E; red) prior to application of S‐1, ML213, NS11021, pinacidil and nicardipine as seen in Figures 1, 2 and 3. All values are expressed as means ± SEM error bars. A 2‐way statistical ANOVA with a post‐hoc Bonferroni test was used to generate significance values. (*n*=) number of animals used.
**Figure S4:** Oestrus cycle dependent differences in U46619‐mediated contraction in mesenteric, cerebral, and coronary arteries.
Mean data of contraction in response to U46619 (0.003‐3 μmol‐L‐1) within mesenteric (n = 8–10; A, cerebral (n = 5–7; B) and coronary (n = 4–7; C) arteries preincubated within DMSO solvent control (Female di‐oestrus/met‐oestrus (D/M), blue; Female pro‐oestrus/oestrus (P/E), red) or Linopirdine (10 μmol‐L‐1; Female D/M, blue‐dashed line; Female P/E, red‐dashed line). Scatter graph representing the raw EC50 values of U46619‐mediated contraction within cerebral (C) and coronary (D) arteries of the mean data above, in addition to vessels pre‐incubated in KV7.1 specific blocker HMR‐1556 (10 μmol‐L‐1; grey). A two‐way statistical ANOVA with a post‐hoc Bonferroni (A,B,C) or Dunnet's (C,D,E) correction was used to generate significance values (*/#/$ = P ≤ 0.05). Post‐hoc Bonferroni statistical comparisons include Ctrl v Ctrl (* = D/M DMSO v P/E DMSO), Ctrl v Same group condition (# = D/M DMSO v D/M 10 μmol‐L‐1 Linopirdine) and Ctrl v Different group condition ($ = D/M DMSO v P/E 10 μmol‐L‐1 Linopirdine; A,B). Dunnet's statistical comparisons include (* = DMSO v Condition, D). (n=) number of animals used (A‐D).
**Figure S5:** Oestrus cycle dependent differences in Isoprenaline‐mediated relaxation in mesenteric, cerebral and coronary arteries
Mean data of relaxation in response to isoprenaline (0.003‐3 μmol‐L‐1) within mesenteric (n = 8–9; A), cerebral (n = 5–7; B) and coronary (n = 4–7; C) arteries preincubated within DMSO solvent control (Female di‐oestrus/met‐oestrus (D/M), blue; Female pro‐oestrus/oestrus (P/E), red) or Linopirdine (10 μmol‐L‐1; Female D/M, blue‐dashed line; Female P/E, red‐dashed line). Scatter graph representing the raw EC50 values of U46619‐mediated contraction within mesenteric (C), cerebral (D) and coronary (E) arteries of the mean data above, in addition to vessels pre‐incubated in KV7.1 specific blocker HMR‐1556 (10 μmol‐L‐1; grey). A two‐way statistical ANOVA with a post‐hoc Bonferroni (A,B,C) or Dunnet's (C,D,E) correction was used to generate significance values (*/#/$ = P ≤ 0.05). Post‐hoc Bonferroni statistical comparisons include Ctrl v Ctrl (* = D/M DMSO v P/E DMSO), Ctrl v Same group condition (# = D/M DMSO v D/M 10 μmol‐L‐1 Linopirdine) and Ctrl v Different group condition ($ = D/M DMSO v P/E 10 μmol‐L‐1 Linopirdine; A,B). Dunnet's statistical comparisons include (* = DMSO v Condition, D). (n=) number of animals used (A‐F).
**Figure S6:** Relative mRNA transcript for *Kcnq1–5* and *Kcne1–5* within arteries from female (P/E2) and female (D/M) Wistar rats
Relative transcript abundance for *Kcnq1–5* and *Kcne1–5* were measured within renal (*n* = 4–5; A,B) and mesenteric arteries (*n* = 4–5; C,D) and heart (E,F; *n* = 4) and brain (G,H; *n* = 4) from female pro‐oestrus/oestrus (P/E; red) and female di‐oestrus/met‐oestrus (D/M; blue) and mixed female (Grey) Wistar rats when compared to appropriate reference genes (2^−ΔCq^) included the following; renal (*Top1, Ubc*) and mesenteric (*Canx, Cyc*1), heart *(Cyc1),* brain (*Gapdh*). All values are expressed as means ± SEM error bars. A 2‐way statistical ANOVA with a post‐hoc Bonferroni test was used to generate significance values. (*n*=) number of animals used.
**Figure S7:** Immunocytochemistry of K_V_7.1, K_V_7.5 and KCNE4 in isolated renal artery vascular smooth muscle cells from female Wistars.
Representative images of immunocytochemistry demonstrates K_V_7.1 (A,D), K_V_7.5 (B,E) and KCNE4 (C,F) staining (red) from female di‐oestrus/met‐oestrus (D/M; A‐C; *n* = 3) and female pro‐oestrus/oestrus (P/E; D‐F; *n* = 3) in isolated renal artery vascular smooth muscle cells. Plasma membrane and nuclear markers, wheat germ agglutinin (WGA; green) and 4′,6‐diamidino‐2‐phenylindole (DAPI; blue) respectively, are also shown. Insets demonstrate separated target protein (K_V_7.1, K_V_7.5, K4) and membrane marker (WGA). Mean data for Membrane:Cytosol ratio (B) and total cell fluorescence measured in arbitrary units (A.U; C)*.* All values are expressed as mean ± SEM. n = number of animals used. 10–15 cells per n, shading represents individual rats.
**Figure S8:** Relative mRNA transcript for *Esr1, Esr2* and *Gper1* within arteries from female P/E and female D/M Wistar rats.
Relative transcript abundance for *Esr1, Esr2* and *Gper1* were measured within within mesenteric (*n* = 5; A) and renal arteries (*n* = 5; B) and uterus from female pro‐oestrus/oestrus (P/E; red) and female di‐oestrus/met‐oestrus (D/M; blue) and mixed females (*n* = 4; Grey; C) Wistar rats when compared to appropriate reference genes (2^−ΔCq^) included the following; mesenteric (*Canx, Cyc1*), renal (*Top1, Ubc*) and uterus *(Cyc1, Canx*). (*n*=) number of animals used.
**Figure S9:** Oestradiol E2 mediated inhibition of K_V_7 activator mediated relaxation is time dependent.
Mean data for ML213 mediated relaxation (0.01–10 μmol‐L^−1^) of pre‐contracted arterial tone in (U46619; 300 nmol‐L^−1^) in renal (A,B *n* = 5) and mesenteric (C,D; *n* = 5–8) arteries from female D/M (A,C,D) and female P/E (B,D,F) Wistars pre‐incubated in solvent control (DMSO/Ethanol; blue / red), Oestradiol E2 (0.01 μmol‐L^−1^; E2) pre‐incubated for 5 mins (black), or 30 mins (grey, dashed line). Mean data for ML213 mediated relaxation of pre‐contracted arterial tone in mesenteric arteries from female D/M and female P/E Wistars pre‐incubated in solvent control for 4 hrs (blue/red) or 10 nmol‐L^−1^ E2 for 4 hrs (black) or 10 mins, then washed and left for 4 h (grey). All values are expressed as means ± SEM error bars. A 2‐way statistical ANOVA with a post‐hoc Dunnet's correction was used to generate significance values (*/# = *P* ≤ 0.05; * = Ethanol v 30 min E2; # = Ethanol v 5 min E2; A‐D; * = Ethanol v 4 hr E2; $ = Ethanol v 4 Hr E2 (wash);E,F). (*n=*) number of animals used.
**Figure S10:** Oestradiol E2 incubation has no effect on K_V_ 7.4 membrane abundance in isolated mesenteric artery vascular smooth muscle cells from Female P/E Wistar rats.
Representative images of immunocytochemistry demonstrates K_V_7.4 expression (red) from female pro‐oestrus/oestrus (P/E; *n* = 3) mesenteric artery vascular smooth muscle cells pre‐incubated in either solvent control (ethanol/DMSO), Oestradiol (E2; 10 nmol‐L^−1^) or G‐1 (1 μmol‐L^−1^; A) for 30 min prior to fixing. Plasma membrane and nuclear markers wheat germ agglutinin (WGA; green) and 4′,6‐diamidino‐2‐phenylindole (DAPI; blue) are also shown. Fluorescence intensity profiles were plotted for K_V_7.4 and WGA measured in arbitrary units (A.U) along the yellow line seen in the merged image above. Fluorescence intensity ≥200 A. U was considered the plasma membrane (M) and below the threshold was considered the cytosol (C). Bar chart demonstrating mean data of the Membrane:Cytosol ratio for K_V_7.4 expression solvent control (red), E2 (grey) or G‐1 (grey, square pattern; C). Membrane:Cytosol ratio for K_V_7.4 expression was calculated by measuring the fluorescence intensity of K_V_7.4 within the membrane and dividing it by the fluorescence intensity of K_V_7.4 within cytosol from three randomly drawn lines. All values are expressed as mean ± SEM. n = number of animals used. 8‐12 cells per n, shading represents individual rats.
**Figure S11:** E2 mediated effects on ion channel modulators in male mesenteric arteries.
Mean data for ML213 (0.01–10 μmol‐L‐1; A) and NS11021 (0.1–10 μmol‐L‐1; B) mediated relaxation of pre‐contracted arterial tone in (U46619; 300 nmol‐L^−1^) in mesenteric arteries from male (*n* = 5) Wistars pre‐incubated in solvent control (DMSO/Ethanol; black), E2 (Grey; 10 nmol‐L‐1; A,B) and G‐1 (Grey, dashed line; A). All values are expressed as means ± SEM error bars. A 2‐way statistical ANOVA with a post‐hoc Dunnet's (A) or Bonferroni (B) correction was used to generate significance values. (*n=*) number of animals used.
**Figure S12:** Progesterone has no effect on ML213 mediated relaxation.
Mean data for ML213 mediated relaxation (0.01–10 μmol‐L^−1^) of pre‐contracted arterial tone in (U46619; 300 nmol‐L^−1^) in mesenteric arteries from female Di‐oestrus /Met‐oestrus (F‐D/M; *n* = 6–8*;* A) and female Pro‐oestrus/Oestrus (F‐P/E; *n* = 5–6; B) Wistars pre‐incubated in solvent control (DMSO/Ethanol; blue F‐D/M/red F‐P/E), Progesterone (0.01 μmol‐L^−1^; P4) pre‐incubated for 5 mins (black) or 30 mins (grey). All values are expressed as means ± SEM error bars. A 2‐way statistical ANOVA with a post‐hoc Dunnet's correction was used to generate significance values. (*n=*) number of animals used.

## Data Availability

The data generated herein are available upon reasonable request to the corresponding author.
