# Supplementary material for: Marked oestrous cycle‐dependent regulation of rat arterial KV7.4 channels driven by GPER1
Source: Br J Pharmacol. 2022 Oct 11;180(2):174–93. doi: 10.1111/bph.15947 (PMC10091994; doi:10.1111/bph.15947)
Supplement: Supplementary file 1 — Figure S1: Cervical histology throughout the oestrus cycle. Representative images of cell suspension lifted from the cervix during met‐oestrus (Ai/Aii), di‐oestrus (Bi/Bii), pro‐oestrus (Ci/Cii) and oestrus (Di/Dii) at magnifications x10 (Ai‐Di), x20 (Aii,Cii,Dii) and x40 (Bii) and stained in toluidine blue. Arrows demonstrate nucleated epithelial cells (orange), anucleated epithelial cells (blue) and leukocytes (green; Aii‐Dii). Grey arrow demonstrates a ‘swirl’ of nucleated epithelial cells typical of pro‐oestrus, used as an additive tool in determining cycle stage (Cii). Figure S2: Validating Anti‐KV7.4 #APC‐164. Chinese hamster ovarian (CHO) cells transfected with plasmids containing Kcnq4 presented with diffuse labelling for KV7.4 (A; red). Comparably, no staining was observed in Kcnq4‐transfected CHO cells supplemented with blocking peptide (#BLP‐PC164; B) nor non‐transfected CHO cells (C). Similarly, male mesenteric artery (MA) vascular smooth muscle cells presented with diffuse labelling for KV7.4 (D), but not when supplemented with blocking peptide (#BLP‐PC164; E) nor in in the absence of a primary antibody (Noº Ab control; F). 4′,6‐diamidino‐2‐phenylindole (DAPI; blue; A‐F), wheat germ agglutinin membrane marker (green) merged image (merge; D‐F) insets. Figure S3: Pre‐contracted arterial tone in arteries from female rats prior to application of ion channel modulators. Mean data and scatter plot for stable ΔmN in response to 300 nmol‐L−1 U46619 in renal (A) and mesenteric (B) arteries taken from females in Di‐oestrus (F‐D/M; blue) and females in Pro‐oestrus and Oestrus (F‐P/E; red) prior to application of S‐1, ML213, NS11021, pinacidil and nicardipine as seen in Figures 1, 2 and 3. All values are expressed as means ± SEM error bars. A 2‐way statistical ANOVA with a post‐hoc Bonferroni test was used to generate significance values. (n=) number of animals used. Figure S4: Oestrus cycle dependent differences in U46619‐mediated contraction in mesenteric, cerebr [file BPH-180-174-s001.pdf]

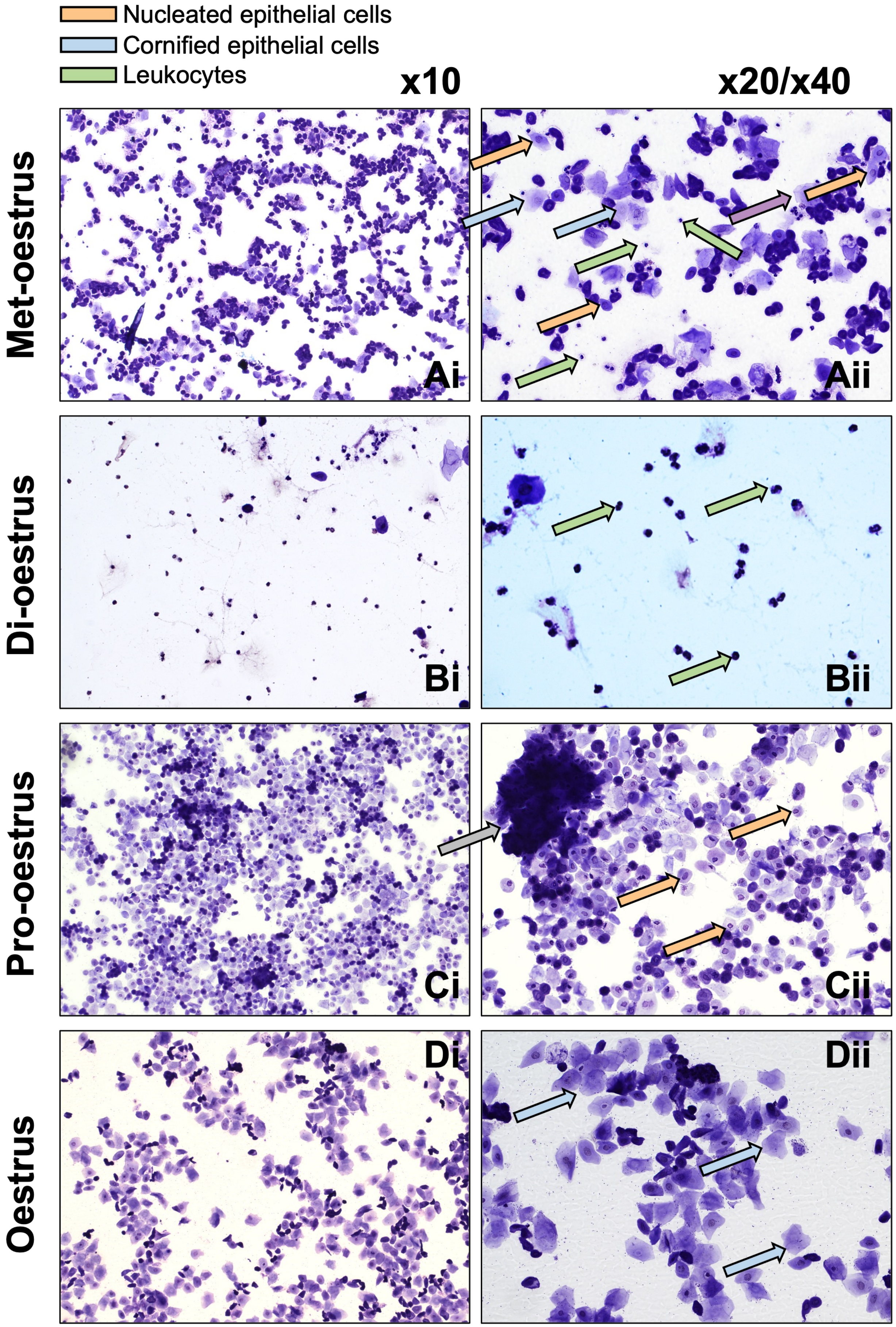

Figure S1: Cervical histology throughout the oestrus cycle.

Representative images of cell suspension lifted from the cervix during met-oestrus (Ai/Aii), di-oestrus (Bi/Bii), pro-oestrus (Ci/Cii) and oestrus (Di/Dii) at magnifications x10 (Ai-Di), x20 (Aii,Cii,Dii) and x40 (Bii) and stained in toluidine blue. Arrows demonstrate nucleated epithelial cells (orange), anucleated epithelial cells (blue) and leukocytes (green; Aii-Dii). Grey arrow demonstrates a 'swirl' of nucleated epithelial cells typical of pro-oestrus, used as an additive tool in determining cycle stage (Cii).

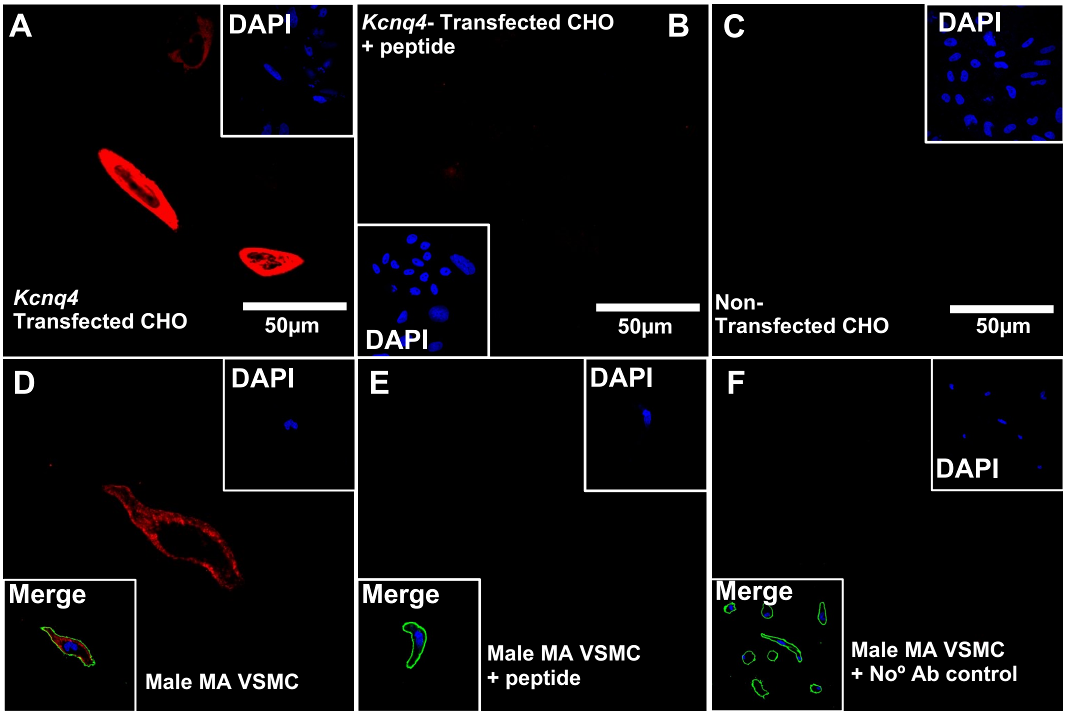

Figure S2: Validating Anti-K_V7.4 #APC-164.

Chinese hamster ovarian (CHO) cells transfected with plasmids containing *Kcnq4* presented with diffuse labelling for K_V7.4 (A; red). Comparably, no staining was observed in *Kcnq4*-transfected CHO cells supplemented with blocking peptide (#BLP-PC164; B) nor non-transfected CHO cells (C). Similarly, male mesenteric artery (MA) vascular smooth muscle cells presented with diffuse labelling for K_V7.4 (D), but not when supplemented with blocking peptide (#BLP-PC164; E) nor in in the absence of a primary antibody (No° Ab control; F). 4′,6-diamidino-2-phenylindole (DAPI; blue; A-F), wheat germ agglutinin membrane marker (green) merged image (merge; D-F) insets.

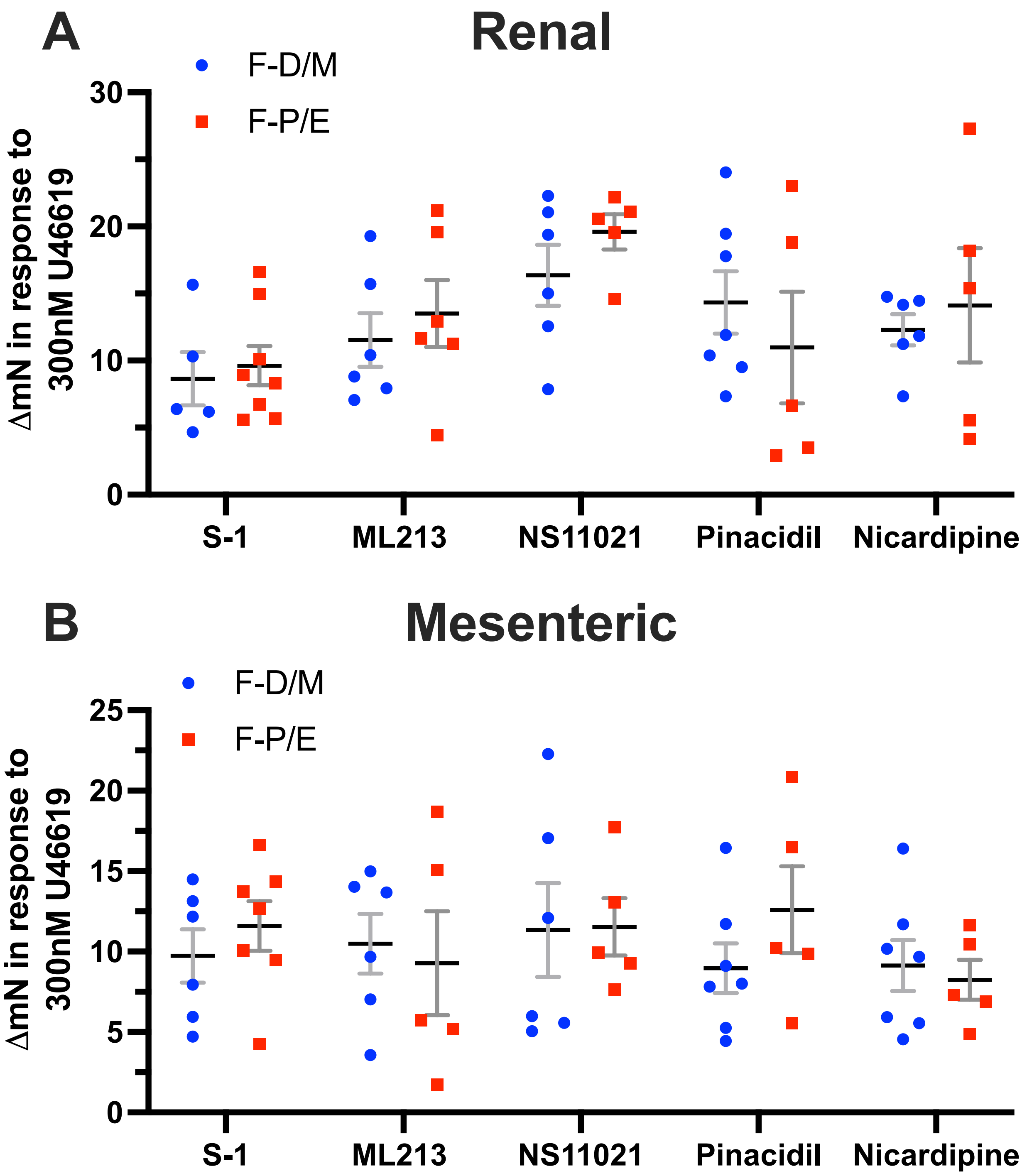

Figure S3: Pre-contracted arterial tone in arteries from female rats prior to application of ion channel modulators.
Mean data and scatter plot for stable ΔmN in response to 300 nmol-L^{-1} U46619 in renal (A) and mesenteric (B) arteries taken from females in Di-oestrus (F-D/M; blue) and females in Pro-oestrus and Oestrus (F-P/E; red) prior to application of S-1, ML213, NS11021, pinacidil and nicardipine as seen in Figures 1, 2 and 3. All values are expressed as means ± SEM error bars. A 2-way statistical ANOVA with a post-hoc Bonferroni test was used to generate significance values. (*n*=) number of animals used.

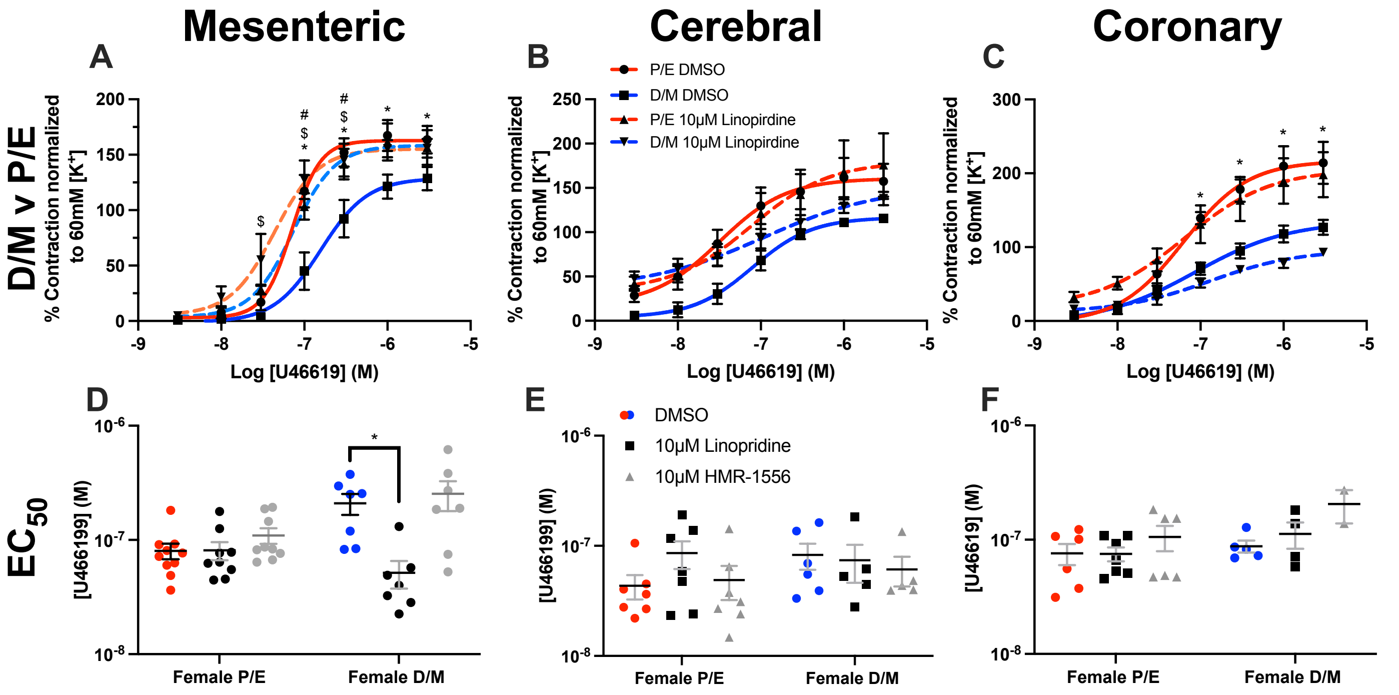

Figure S4: Oestrus cycle dependent differences in U46619-mediated contraction in mesenteric, cerebral and coronary arteries.

Mean data of contraction in response to U46619 (0.003-3μmol-L^{-1}) within mesenteric (*n*=8-10; A, cerebral (*n*=5-7; B) and coronary (*n*=4-7; C) arteries preincubated within DMSO solvent control (Female di-oestrus/met-oestrus (D/M), blue; Female pro-oestrus/oestrus (P/E), red) or Linopirdine (10μmol-L^{-1}; Female D/M, blue-dashed line; Female P/E, red-dashed line). Scatter graph representing the raw EC_{50} values of U46619-mediated contraction within cerebral (C) and coronary (D) arteries of the mean data above, in addition to vessels pre-incubated in $K_V7.1$ specific blocker HMR1556 (10μmol-L^{-1}; grey). A two-way statistical ANOVA with a post-hoc Bonferroni (A,B,C) or Dunnet's (C,D,E) correction was used to generate significance values (*/#/$= P≤0.05). Post-hoc Bonferroni statistical comparisons include Ctrl v Ctrl (*= D/M DMSO v P/E DMSO), Ctrl v Same group condition (#= D/M DMSO v D/M 10μmol-L-1 Linopirdine) and Ctrl v Different group condition ($= D/M DMSO v P/E 10μmol-L-1 Linopirdine; A,B). Dunnet's statistical comparisons include (*= DMSO v Condition, D). (n=) number of animals used (A-D).

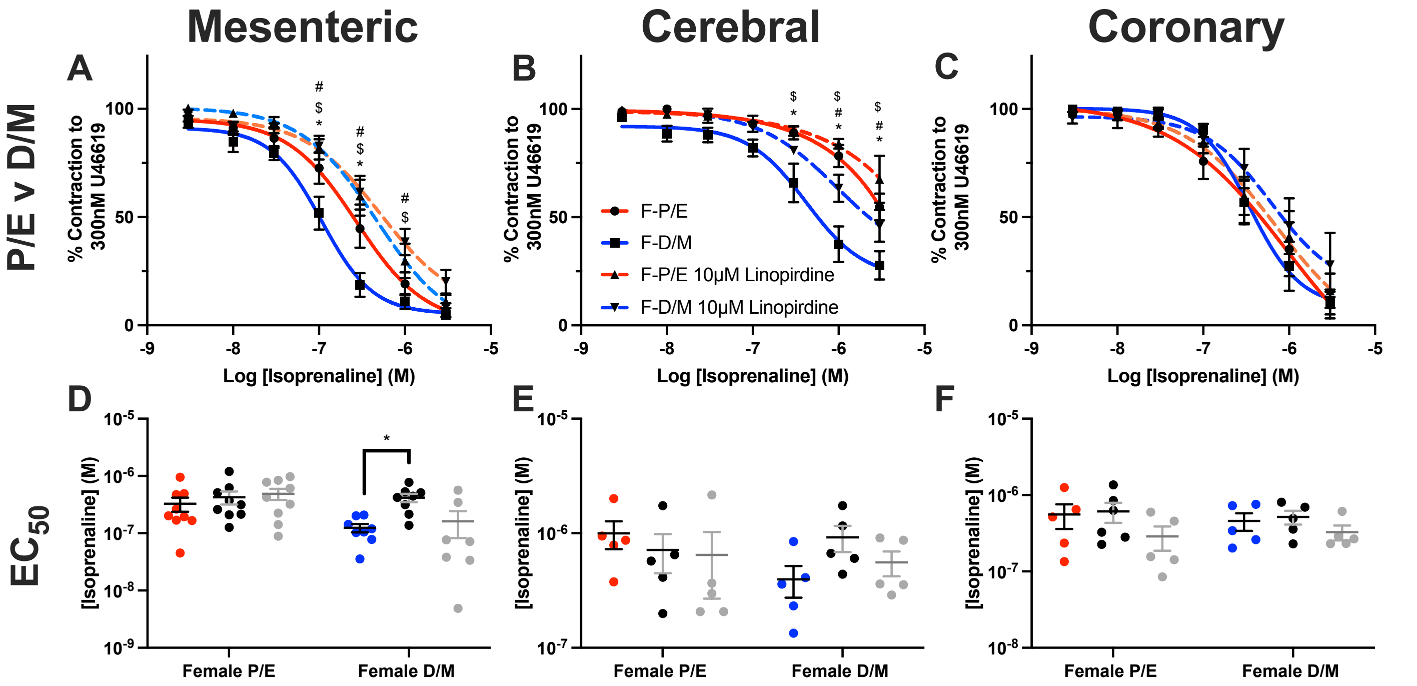

Figure S5: Oestrus cycle dependent differences in Isoprenaline-mediated relaxation in mesenteric, cerebral and coronary arteries

Mean data of relaxation in response to isoprenaline (0.003-3μmol-L^{-1}) within mesenteric (*n*=8-9; A), cerebral (*n*=5-7; B) and coronary (*n*=4-7; C) arteries preincubated within DMSO solvent control (Female di-oestrus/met-oestrus (D/M), blue; Female pro-oestrus/oestrus (P/E), red) or Linopirdine (10μmol-L^{-1}; Female D/M, blue-dashed line; Female P/E, red-dashed line). Scatter graph representing the raw EC_{50} values of U46619-mediated contraction within mesenteric (C), cerebral (D) and coronary (E) arteries of the mean data above, in addition to vessels pre-incubated in K_V7.1 specific blocker HMR1556 (10μmol-L^{-1}; grey). A two-way statistical ANOVA with a post-hoc Bonferroni (A,B,C) or Dunnet's (C,D,E) correction was used to generate significance values (*/#/$= P≤0.05). Post-hoc Bonferroni statistical comparisons include Ctrl v Ctrl (*= D/M DMSO v P/E DMSO), Ctrl v Same group condition (#= D/M DMSO v D/M 10μmol-L-1 Linopirdine) and Ctrl v Different group condition ($= D/M DMSO v P/E 10μmol-L-1 Linopirdine; A,B). Dunnet's statistical comparisons include (*= DMSO v Condition, D). (*n*=) number of animals used (A-F).

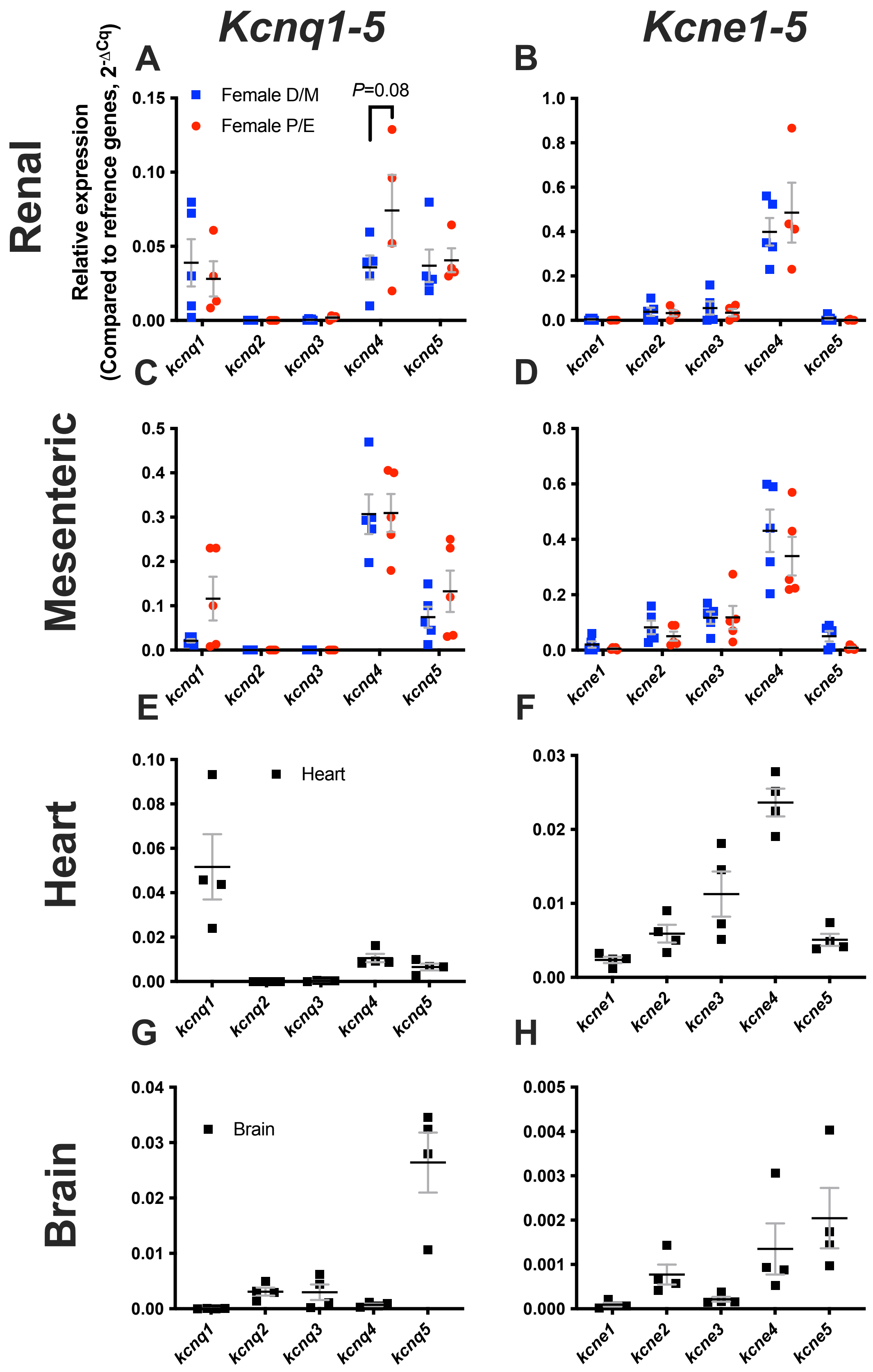

Figure S6: Relative mRNA transcript for *Kcnq1-5* and *Kcne1-5* within arteries from female (P/E2) and female (D/M) Wistar rats

Mean data and scatter plot for relative transcript abundance for *Kcnq1-5* and *Kcne1-5* were measured within renal (*n*=4-5; A,B) and mesenteric arteries (*n*=4-5; C,D) and heart (E,F; *n*=4) and brain (G,H; *n*=4) from female pro-oestrus/oestrus (P/E; red) and female di-oestrus/met-oestrus (D/M; blue) and mixed female (Grey) Wistar rats when compared to appropriate reference genes ($2^{-\Delta Cq}$) included the following; renal (*Top1, Ubc*) and mesenteric (*Canx, Cyc*1), heart *(Cyc1),* brain (*Gapdh*). All values are expressed as means ± SEM error bars. A 2-way statistical ANOVA with a post-hoc Bonferroni test was used to generate significance values. (*n*=) number of animals used.

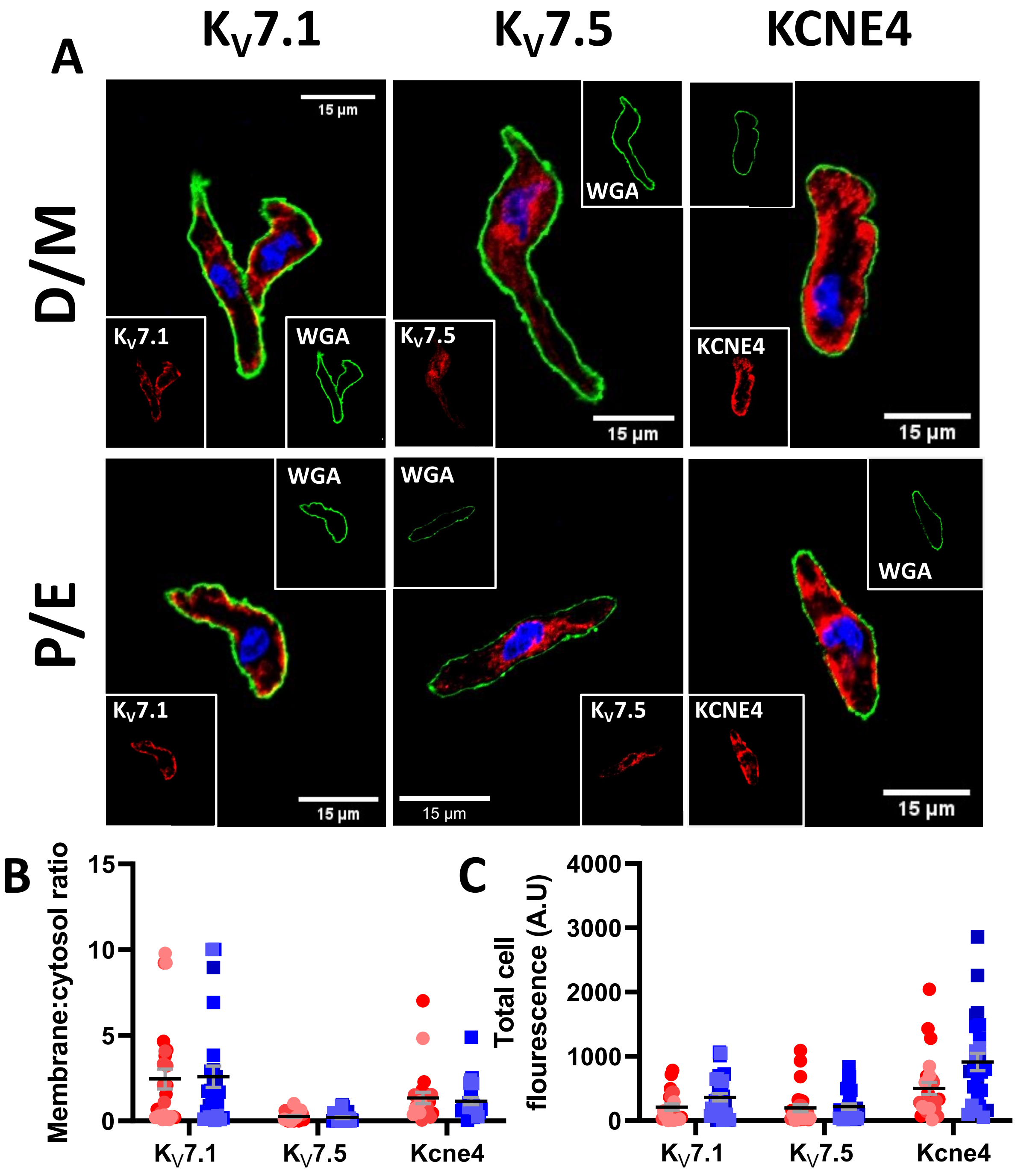

Figure S7: Immunocytochemistry of $K_V7.1$, $K_V7.5$ and KCNE4 in isolated renal artery vascular smooth muscle cells from female Wistars.

Representative images of immunocytochemistry demonstrates $K_V7.1$ (A,D), $K_V7.5$ (B,E) and KCNE4 (C,F) staining (red) from female di-oestrus/met-oestrus (D/M; A-C; *n*=3) and female pro-oestrus/oestrus (P/E; D-F; *n*=3) in isolated renal artery vascular smooth muscle cells. Plasma membrane and nuclear markers, wheat germ agglutinin (WGA; green) and 4′,6-diamidino-2-phenylindole (DAPI; blue) respectively, are also shown. Insets demonstrate separated target protein ($K_V7.1$, $K_V7.5$, K4) and membrane marker (WGA). Mean data and scatter plot for Membrane:Cytosol ratio (B) and total cell fluorescence measured in arbitrary units (A.U; C). All values are expressed as means ± SEM error bars. (*n*=) number of animals used, 8-12 cells per *n*.

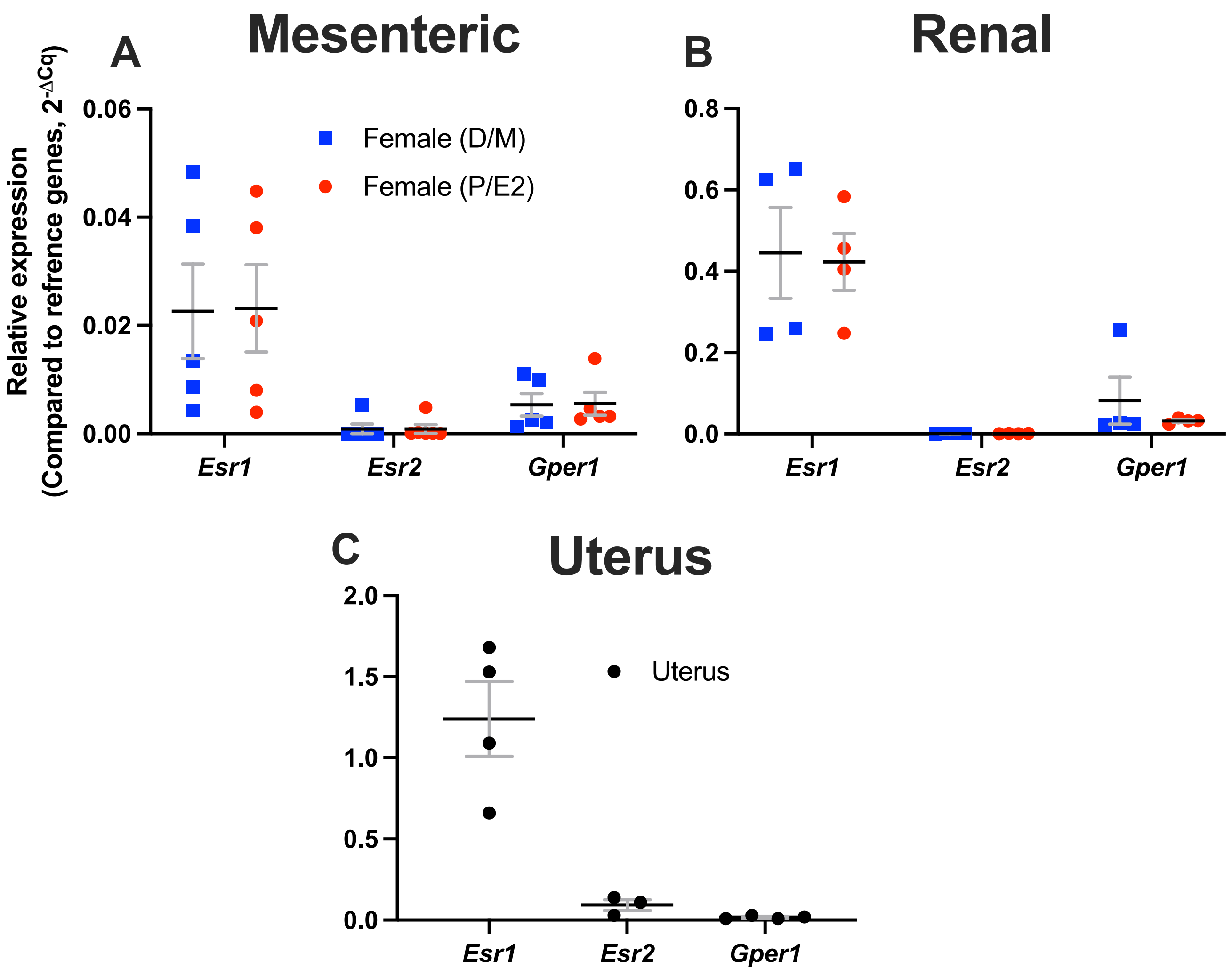

Figure S8: Relative mRNA transcript for *Esr1, Esr2* and *Gper1* within arteries from female P/E and female D/M Wistar rats.

Relative transcript abundance for *Esr1, Esr2* and *Gper1* were measured within within mesenteric (*n*=5; A) and renal arteries (*n*=4; B) and uterus from female pro-oestrus/oestrus (P/E; red) and female di-oestrus/met-oestrus (D/M; blue) and mixed females (*n*=4; Grey; C) Wistar rats when compared to appropriate reference genes ($2^{-\Delta Cq}$) included the following; mesenteric (*Canx, Cyc1*), renal (*Top1, Ubc*) and uterus *(Cyc1, Canx*). (*n*=) number of animals used.

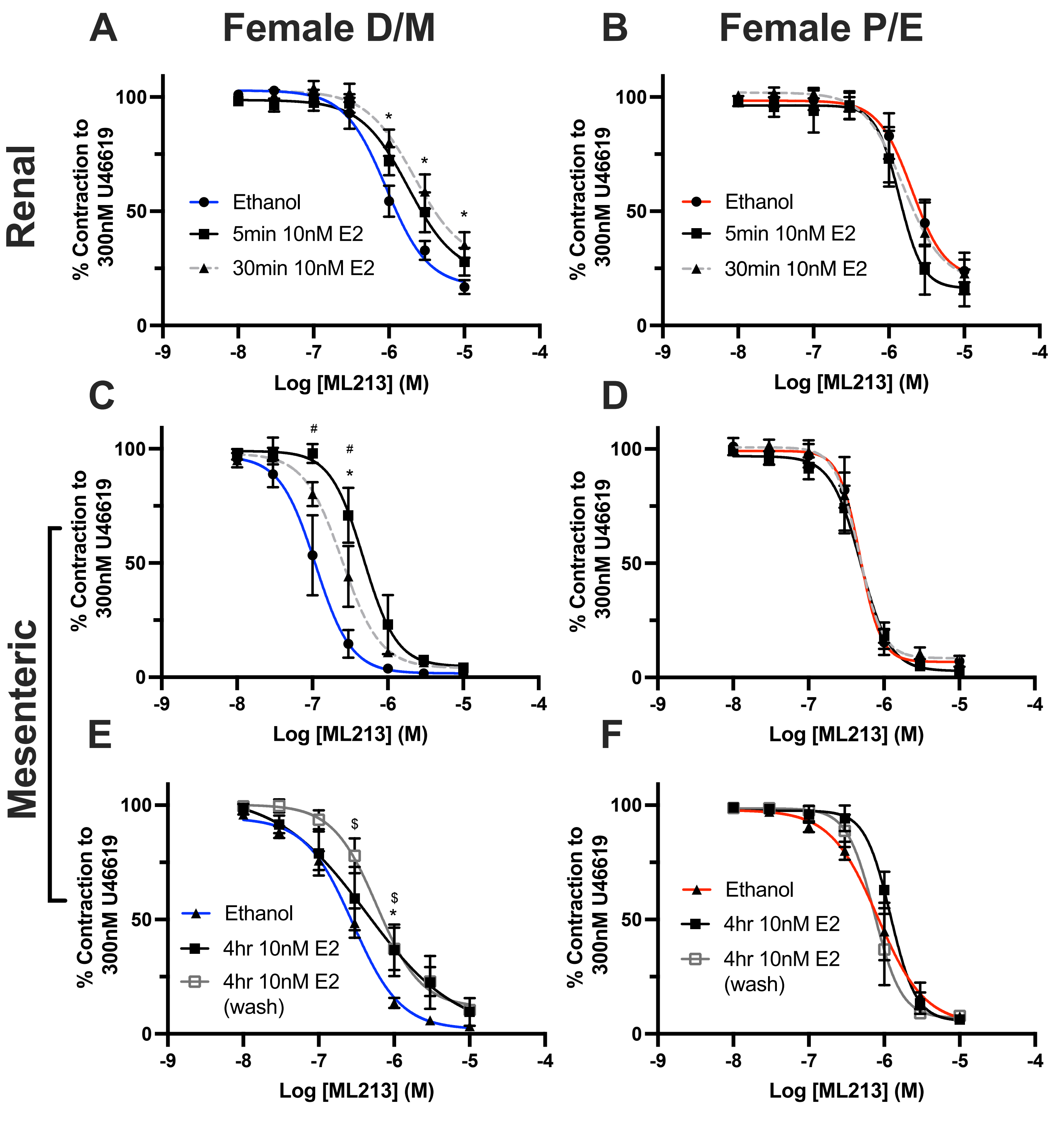

Figure S9: Oestradiol E2 mediated inhibition of K_V7 activator mediated relaxation is time dependent.

Mean data for ML213 mediated relaxation (0.01-10 μmol-L^{-1}) of pre-contracted arterial tone in (U46619; 300 nmol-L^{-1}) in renal (A,B *n*=5) and mesenteric (C,D; *n*= 5-8) arteries from female D/M (A,C,D) and female P/E (B,D,F) Wistars pre-incubated in solvent control (DMSO/Ethanol; blue / red), Oestradiol E2 (0.01μmol-L^{-1}; E2) pre-incubated for 5mins (black), or 30mins (grey, dashed line). Mean data for ML213 mediated relaxation of pre-contracted arterial tone in mesenteric arteries from female D/M and female P/E Wistars pre-incubated in solvent control for 4 hrs (blue/red) or 10 nmol-L^{-1} E2 for 4 hrs (black) or 10 mins, then washed and left for 4 hrs (grey). All values are expressed as means ± SEM error bars. A 2-way statistical ANOVA with a post-hoc Dunnet's correction was used to generate significance values (*/#= *P*≤0.05; *= Ethanol v 30min E2; #= Ethanol v 5min E2; A-D; *=Ethanol v 4 hr E2; $= Ethanol v 4 Hr E2 (wash);E,F). (*n*=) number of animals used.

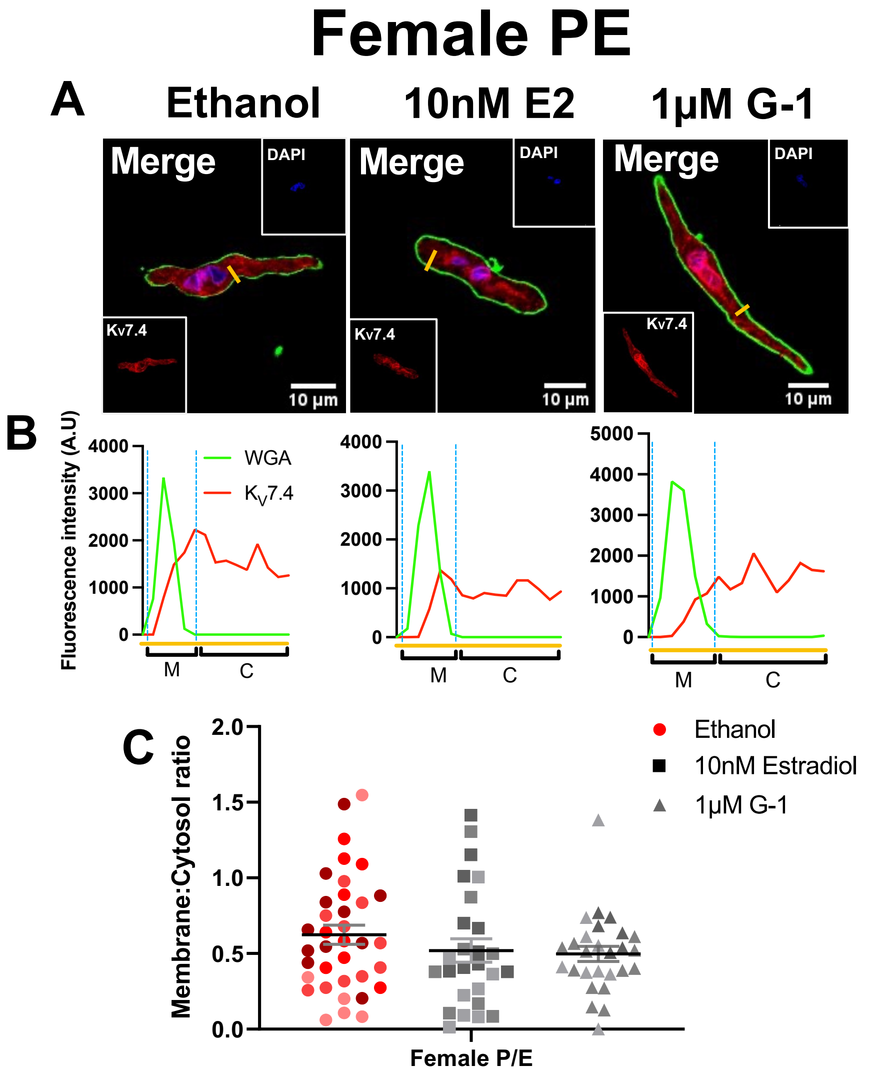

Figure S10: Oestradiol E2 incubation has no effect on K_V7.4 membrane abundance in isolated mesenteric artery vascular smooth muscle cells from Female P/E Wistar rats.

Representative images of immunocytochemistry demonstrates K_V7.4 expression (red) from female pro-oestrus/oestrus (P/E; *n*=3) mesenteric artery vascular smooth muscle cells pre-incubated in either solvent control (ethanol/DMSO), Oestradiol (E2; 10 nmol-L^{-1}) or G-1 (1 µmol-L^{-1}; A) for 30 min prior to fixing. Plasma membrane and nuclear markers wheat germ agglutinin (WGA; green) and 4′,6-diamidino-2-phenylindole (DAPI; blue) are also shown. Fluorescence intensity profiles were plotted for K_V7.4 and WGA measured in arbitrary units (A.U) along the yellow line seen in the merged image above. Fluorescence intensity ≥200 A.U was considered the plasma membrane (M) and below the threshold was considered the cytosol (C). Bar chart demonstrating mean data of the Membrane:Cytosol ratio for K_V7.4 expression solvent control (red), E2 (grey) or G-1 (grey, square pattern; C). Membrane:Cytosol ratio for K_V7.4 expression was calculated by measuring the fluorescence intensity of K_V7.4 within the membrane and dividing it by the fluorescence intensity of K_V7.4 within cytosol from three randomly drawn lines in 10-12 cells pre *n.* All values are expressed as means ± SEM error bars. A 2-way statistical ANOVA with a post-hoc Dunnet's correction was used to generate significance values. (*n*=) number of animals used.

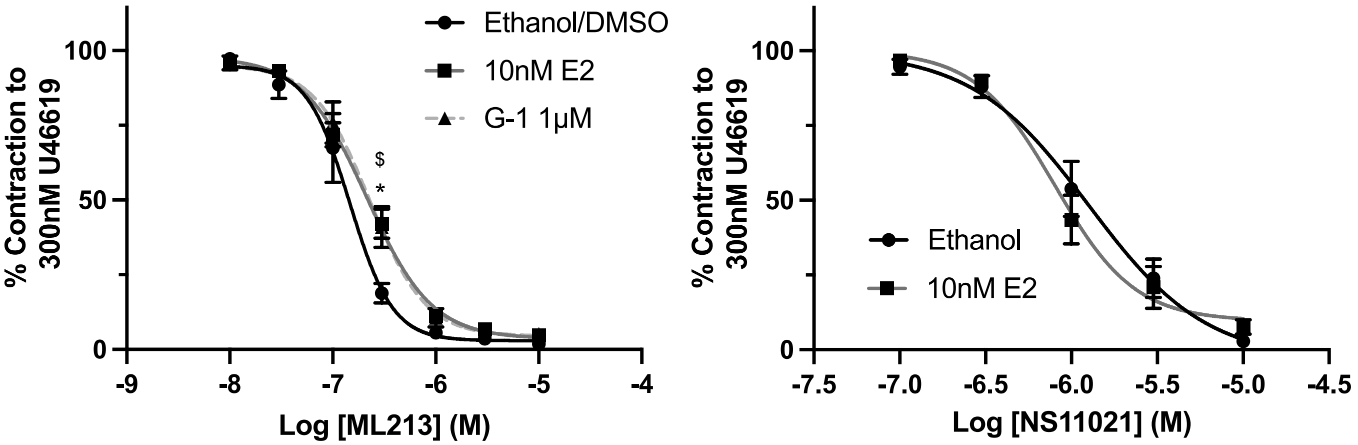

Figure S11: E2 mediated effects on ion channel modulators in male mesenteric arteries.

Mean data for ML213 (0.01-10 μmol-L-1; A) and NS11021 (0.1-10 μmol-L-1; B) mediated relaxation of pre-contracted arterial tone in (U46619; 300 nmol-L^{-1}) in mesenteric arteries from male (*n*=5) Wistars pre-incubated in solvent control (DMSO/Ethanol; black), E2 (Grey; 10 nmol-L-1; A,B) and G-1 (Grey, dashed line; A). All values are expressed as means ± SEM error bars. A 2-way statistical ANOVA with a post-hoc Dunnet's (A) or Bonferroni (B) correction was used to generate significance values. (*n*=) number of animals used.

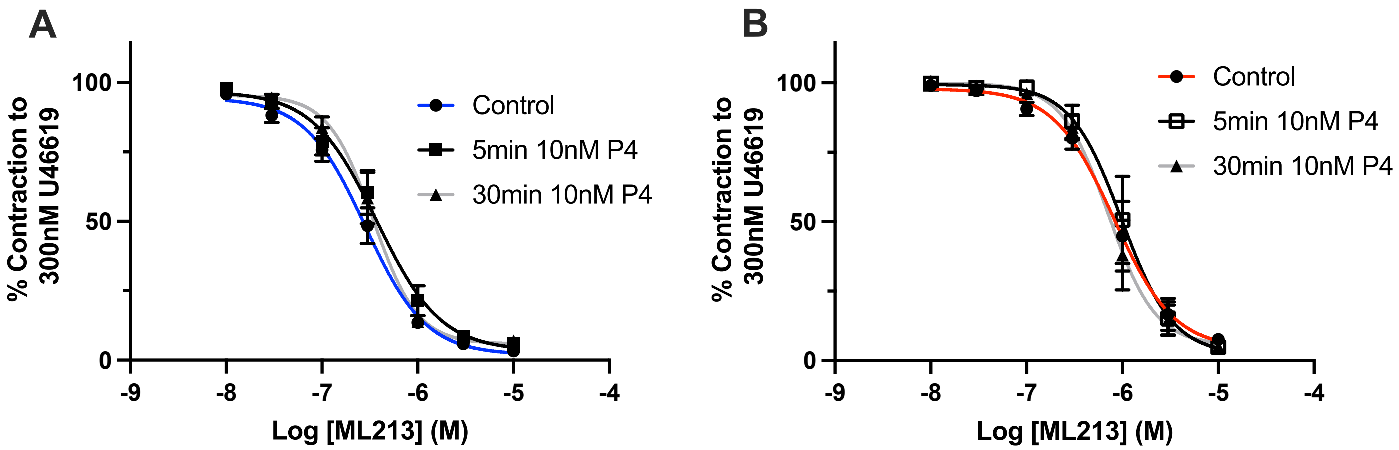

Figure S12: Progesterone has no effect on ML213 mediated relaxation.

Mean data for ML213 mediated relaxation (0.01-10 µmol-L^{-1}) of pre-contracted arterial tone in (U46619; 300 nmol-L^{-1}) in mesenteric arteries from female Di-oestrus / Met-oestrus (F-D/M; *n*=6-8*;* A) and female Pro-oestrus / Oestrus (F-P/E; *n*=5-6; B) Wistars pre-incubated in solvent control (DMSO/Ethanol; blue F-D/M / red F-P/E), Progesterone (0.01 µmol-L^{-1}; P4) pre-incubated for 5 mins (black) or 30mins (grey). All values are expressed as means ± SEM error bars. A 2-way statistical ANOVA with a post-hoc Dunnet's correction was used to generate significance values. (*n*=) number of animals used.
